# Scholarly knowledge graphs through structuring scholarly communication: a review

**DOI:** 10.1007/s40747-022-00806-6

**Published:** 2022-08-09

**Authors:** Shilpa Verma, Rajesh Bhatia, Sandeep Harit, Sanjay Batish

**Affiliations:** Punjab Engineering College, Chandigarh, India

**Keywords:** Scholarly communication, Knowledge graph construction, Knowledge graph embedding, Utilization

## Abstract

The necessity for scholarly knowledge mining and management has grown significantly as academic literature and its linkages to authors produce enormously. Information extraction, ontology matching, and accessing academic components with relations have become more critical than ever. Therefore, with the advancement of scientific literature, scholarly knowledge graphs have become critical to various applications where semantics can impart meanings to concepts. The objective of study is to report a literature review regarding knowledge graph construction, refinement and utilization in scholarly domain. Based on scholarly literature, the study presents a complete assessment of current state-of-the-art techniques. We presented an analytical methodology to investigate the existing status of *scholarly knowledge graphs* (SKG) by structuring scholarly communication. This review paper investigates the field of applying machine learning, rule-based learning, and natural language processing tools and approaches to construct SKG. It further presents the review of knowledge graph utilization and refinement to provide a view of current research efforts. In addition, we offer existing applications and challenges across the board in construction, refinement and utilization collectively. This research will help to identify frontier trends of SKG which will motivate future researchers to carry forward their work.

## Introduction

With the expansion of academic literature in recent years, retrieving accumulated knowledge from documentation has become a significant problem. Document and keyword-based information retrieval systems are no longer adequate to explore the insights of the scholarly domain. Document-centered scholarly communications contain loads of content to mine, search and recommend. To achieve this criterion, knowledge must be gained through the use of automated tools to utilize the scholarly infrastructure. However, the knowledge presented in scholarly infrastructure resides in the form of text, tables, figures, algorithms, charts, etc., and automatic knowledge curation from these components is not easy due to improper structure. Though scholarly knowledge is ambiguous in nature, the requirement of standard digitalization, organization, and collaborative knowledge representation is an urgent need. In practice, the field of scholarly communication has been fueled by millions of heterogeneous structured and unstructured data resources, which have a high capacity to contain a network of relationships. It is essential to obtain new insights and leverage the organizational structure from the network of scientific knowledge.

**Fig. 1:**
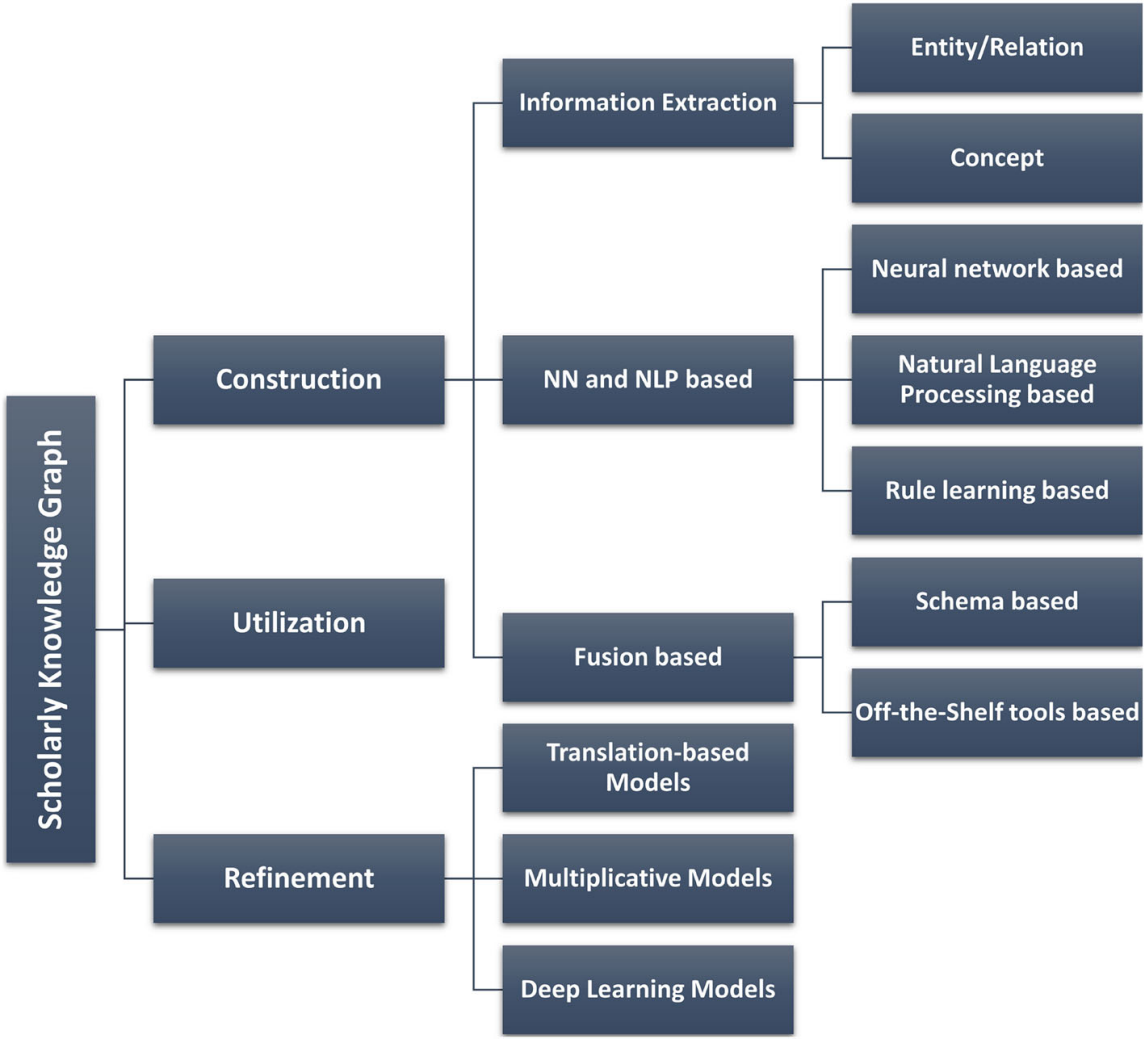
Classification of scholarly knowledge graphs

To accomplish this task, semantic representation provides potential benefits to design structured information systems in the scholarly domain. Semantic representation refers to meaningful concepts present in the field, and richer knowledge can be derived from concepts and relationships. Thus, a semantic model can lead to more prosperous information processing by metadata acquisition, management, publication of scholarly knowledge by applying supervised, unsupervised, and natural language processing techniques. To navigate and discover, semantic technologies involve taxonomy construction, database storage, retrieval, and visualization of the connected scholarly network. Aiming to fill this gap, studies on knowledge graphs build upon scholarly domain are developed, which expresses semantics network and digital objects linking in fewer efforts. Knowledge graphs are useful for determining semantic relatedness by taking into account the hierarchical structure of the scholarly network as well as all forms of semantic relationships between concepts. Knowledge graphs not only measures semantic similarity based on the information gained from large corpora, but also calculates the semantic path distance present between two concepts. Specifically, knowledge-based semantic similarity approaches provide in-depth information about the concepts derived from concept taxonomies. For example, when using a retrieval system to find a certain article, the user’s queries are composed of keywords with a limited query length. In this scenario, analysis of bag-of-words and semantic structure is insufficient to provide an accurate response to a user question. Knowledge graphs combine the capabilities of concept classes and their instances with the help of ontology and capture in-depth semantic relationships to find similar article.

Overall, the notion of knowledge graphs possesses a close connection among semantic web, machine learning, graph databases, and knowledge engineering. Knowledge graphs are a suitable infrastructure to integrate, publish, store, access, and evaluate scholarly semantic communication. Recent advancements in the field of knowledge graph-based representation research focuses on the knowledge acquisition, knowledge graph creation, triple extraction, triple classification, knowledge graph completion. Thus, several real-world applications have been brought into consideration such as collaboration recommendation, scientific community analysis, topic mining, clustering scientific fields, link prediction and automatic creation of scientific document’s components (title, abstract, survey tables, etc.), summarization, hypothesis generation, etc. While analyzing the most important research works and identifying probable future research topics, we focus on more than one aspect depicting in Fig. 1:

**Construction:** Discovering and deriving more that what stated explicitly by leveraging reasoning algorithms for ontologies.

**Refinement:** Representing graph in dense, continuous and low-dimensional vectors to perform machine learning tasks.

**Utilization:** Enabling the graph to be applicable for interactive delivery of results for naive users and stakeholders.

This paper focuses on presenting a current overview of knowledge graph creation in the scholarly area. In the literature, many comprehensive survey papers for knowledge graph [1], domain-specific knowledge graphs such as smart grids [2], industrial products [3], manufacturing and production [4] biomedical domain [5] and knowledge graphs with recommendation engines [6] exist, whereas no survey paper focuses on concept of knowledge graphs in scholarly domain. This motivates us to investigate at the various aspects of the knowledge graph in the scholarly domain and summarize the research findings. To recognize, investigate, and interpret all relevant signals connected to a specific research question, a well-defined approach is employed, which is unbiased and reasonable.To respond to the formulated questions, the data retrieved from the final collection of publications chosen for review was analyzed. The following are the primary contributions of this paper:

- We conduct a review of the knowledge graphs constructed in scholarly domain from the three perspective. The work in the article follows a methodology that provides in-depth detail of the literature focusing on various scholarly knowledge graph construction, utilization and refinement techniques.
- This survey focuses on the construction of KG that further divided into Information extraction (IE), creation method and Schema/ OpenIE tools based integration methods.
- For utilization, graph exploration, querying andvisualization-based studies are covered.
- For refinement, we further divided it into Translation based, Multiplicative and deep learning-based embedding methods that provide the view of triple extracted, task performed, domain used and evaluation method applied.
- We provide wide coverage of many applications such as open knowledge graphs, ranking and recommendations, question answering and academic mining as an emerging applications. Challenges faced during the construction of knowledge graphs are also elaborated.

The remainder of the paper is laid out as follows. Research Questions along with literature search and selection criteria is defined in “Research Methodology”. The background of the large scholarly network domain, the concept of linking knowledge with scholarly communication, and the scholarly domain specific infrastructures are summarised in “Background concepts and open scholarly graphs”. “Knowledge graph construction” describes the process of information extraction focused on scholarly document-centric paradigms and classification of knowledge graph construction techniques. “Knowledge graph utilization in scholarly domain” focuses on the utilization of constructed knowledge graphs that allows the usage and visualization of information. “Knowledge graph refinement” discusses various knowledge graph refinement methods applied to resolve the major challenge of knowledge graph completion. “Scholarly knowledge graph evaluation, ontologies, data models” discusses the evaluation, ontology used and overview of data models. “Scientific knowledge graph application/tasks” and “Future directions/challenges” targets the applications in scholarly knowledge graph domain and summarizes the future directions in this research area respectively. Finally “Conclusion” concludes the paper.

## Research methodology

### Research questions

The emphasis in this study is fully on defining and answering the formulated research questions, as well as exploring the gathered works on scholarly Knowledge graphs from diverse perspectives. Our paper covers three categories horizontally, i.e., knowledge graph construction (KGC), knowledge graph utilization (KGU) and knowledge graph refinement (KGR). Moreover, how KGC, KGU and KGR are divided into categories is mentioned in Table 1 along with the motivation. Our objective is to unravel the research on the topic from various perspectives and conduct the review that is elaborated from the viewpoints of research questions. There are following research questions that can be answered.

**Table 1:** Research questions and motivation

Research questions (RQ)	Motivation
***I. Research studies in scholarly knowledge graph construction (KGC)***	
What type of entities and relationships are extracted during information extraction task?	There is a need to review specific set of entities and relations extracted from literature along with specific domain in order to identify current status in various domains
What approaches have been used for the scholarly knowledge graphs construction?	A most vital step in construction of knowledge graphs in scholarly domain is knowledge extraction completed with the help of extraction tools need to be explored. Along with this, type of knowledge discovery is also an important aspect to cover. The ways of storing and visualize the knowledge graphs to provide various application services is a promising field
What are the ontology and OpenIE tools applied?	It is significant to provide an overview of ontology designed/reused along with Off-the shelf tools applied on scholarly knowledge graphs to exhibit the importance of semantic representation of scholarly communication
***III. Research studies in knowledge graph utilization (KGU)***	
What are the various studies that are deployed and leveraged knowledge graphs as application service?	Various Knowledge graph utilization studies along with link, key features, objective, domain and mappings are important attributes to discuss. This belongs to storing, accessing and updating the required knowledge in suitable output formats
***II. Research studies in scholarly knowledge graph refinement (KGR)***	
What are the application scenarios have been covered in KGR along with embedding approaches used for data completion task?	It is important to analyze the approaches for knowledge graph embedding type, triple type, dataset, evaluation will be covered along with application scenarios in the context of recommendation and data exploration in scholarly domain

### Literature search and selection

An effective search strategy is formed, taking into account a vital pre-requisite, to initiate the survey process through digital libraries to obtain appropriate literature. An automatic search was conducted in this study, taking into account digital libraries such as the ACM Digital Library, Springer, ScienceDirect and IEEE xplore. In addition, Google Scholar also produced a robust base of primary literature relevant to the keywords. Furthermore, for identifying relevant research works, we identified the most prominent conferences such as ISWC, TPDL, WWW, JCDL, CEUR, SAC, CIKM, KDD, to highlight a few. For the first level basic search, we investigated for different keywords for such as “TOPIC=( Knowledge graph) AND (Scholarly OR scientific OR literature OR Academic); Time Span: 2015-2021; Language: English”. 2772 items were found relevant through first round searching and after removal of duplicates, selected articles were narrowed down to 1630. Then a next level search was conducted on title to meet the relevance criteria and 527 articles were filtered. We examined more than 140 research articles refined on the basis of abstract and 70 on the basis of full-text of the paper.

## Background concepts and open scholarly graphs

To draw the relevant ground for our study, a brief introduction to knowledge graphs is provided by summarizing the main steps of its working procedure. In this section, we also introduced the background work from the perspective of large-scale scholarly networks and its linking with semantic resources to obtain scholarly knowledge. In addition, to provide the understandable representation of knowledge graph in scholarly domain, scholarly knowledge graph and its construction workflow is described along with the existing open scholarly graphs.

### Knowledge graph basics

KGs have risen in prominence as a result of a rapid transition from typical linked data and knowledge engineering toward innovative knowledge-based applications. A basic step in laying the foundation for our research is to establish a definition for Knowledge Graphs (KG) as well as key concepts related to knowledge graphs. The term knowledge graphs (KG) first populated in 2012 by Google and many formal definitions have been proposed in the literature [7]. Knowledge graphs are gaining traction in a variety of academic and industrial sectors with expanded concepts, inspired by Google’s shining example. A misleading assumption is that the term knowledge graph is often used interchangeably with knowledge base or ontology. A knowledge graph is generally defined as a data structure that describes concepts and their interactions using a directed, edge labeled graph, often organizing them in an ontological schema. On the web, a number of knowledge graphs have been made available that follow a variety of data representation standards. Along with Google knowledge graph, Freebase, YAGO, NELL, ConceptNet, Wikidata, DBpedia, Facebook’s entity graph, etc. are frequently mentioned in the literature. However, all these implementations differ in architecture, technology used and functionality, making it difficult to reach a consensus and define a knowledge graph.

Based on the basic conceptual analysis, in gensis the key components of knowledge graph explained in below mentioned sections. Some common characteristics are: *Ontology:* Structure of large-scale KG is largely depends on using an ontology that defines a set of concepts with properties and associations across a single or multiple domains. KG provides a common structure allowing various applications to share similar ontology to reuse consisting classes and properties. *Triple:* To obtain compatible data in the form of triple, the infrastructure of KG demands translation of data into RDF that ensures the comprehensible representation of assertion. *Storage:* The creation of the KG involves knowledge curation from structured and unstructured sources containing heterogeneous formats such as CSV, JSON, and XML. *Querying:* As data model heterogeneity is huge, graph DBMS and adaptive querying via different query languages, e.g., Cypher, SPARQL endpoints, SQL and API call is an important step.

**Fig. 2:**
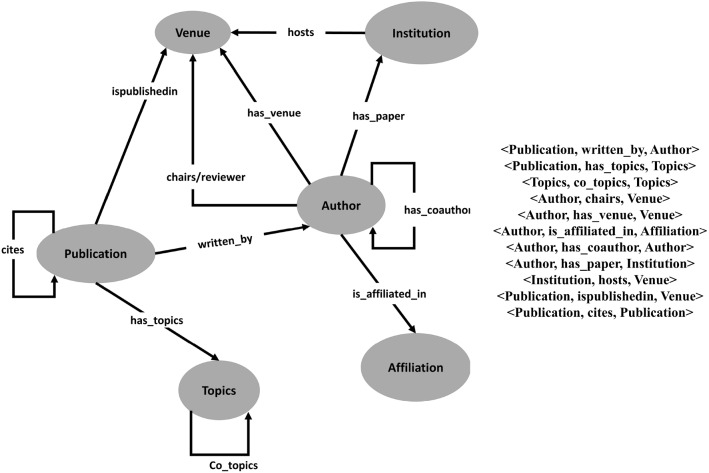
Pictorial view of example of entities/relationships and triples in scholarly knowledge graph (SKG)

### Overview of large-scale scholarly networks and its linking with semantics

In the literature, scholarly communication [8] possessed a long history in the fields of artificial intelligence and information science. The idea of representing scholarly communication in the form of networks is first implemented decade ago as citation networks [9], academic collaboration networks [10], advisor–advisee networks [11, 12], bibliographic coupling networks [13] and many more [14]. In this context, many scholarly data-driven activities such as academic data mining [15], scientific recommendation [16], scholar profiling [17] and scientific impact evaluation [18] have been thoroughly examined. Scholarly documents can be retrieved by crawling and extracting using structural and content-based features [19]. In order to make the data easily discoverable, Digital Object Identifier (DOI) is used to facilitate accessing and traceability. Despite the fact that valid information is easily accessed through the web and open data, generating scholarly network is challenging due to the varied nature of the scholarly data models. The study of scholarly networks entails examining the structural dynamics using data analysis methodologies. Various topological network similarity-based methods such as random-walk [20] and modularity-based topological approaches merely consider the complete set of attributes. Due to the large quantity and dimensionality of scholarly data, traditional graph-based approaches that only deal with structural analysis cannot perform effectively. The network embedding method [21] has lately gained popularity as a method for learning low-dimensional representations of nodes in large networks. Link prediction, node classification, and community discovery are just a few of the network-based applications that have demonstrated its efficacy.

Modern information systems require discovery of structural as well as semantic patterns based knowledge representation of the data model, resulting in a more robust framework for data processing and querying. As a result, several approaches of embedding scholarly semantic information into networks have been developed for a variety of applications. Semantics refers to the structure and meaning of the text in scholarly documents that are hardly accessible and difficult to represent in human-readable format as compared to the character and words. To utilize the hidden semantics between the links in the network, integration of linked open data [22], graph databases and semantic web has been explored. It has been noticed that, challenges such as heterogeneity and scalability have been handled efficiently with the help of linked data sets of scholarly documents. The use of natural language processing (NLP) technologies with notions of URIs, querying the data using RDF and SPARQL, and visualizing results to present them in a more intelligible way are the fundamental cornerstones of semantic scholarly communication. A formal knowledge representation of scholarly data includes creation of graphs supporting representation that is semantically consistent and structured.

**Fig. 3:**
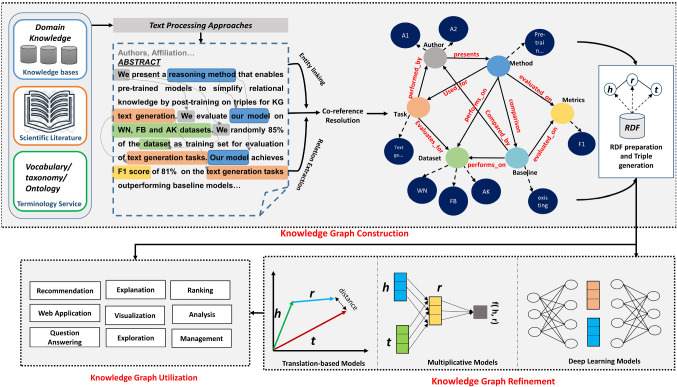
Conceptual view of the process of data mining in scholarly knowledge graphs

### Scholarly knowledge graphs

In most fields, graphs provide a more understandable and concise representation of knowledge. Scholarly Knowledge graph [23] is a semantic directed labeled graph composed of set of entities lined together with relations where nodes represent entities and edges represent relations. A link from a paper to its author, for example, must connect an instance of type $<Publication>$ to an instance of type $<Author>$. All the entities and relationships contains label having semantics which are believed to come from an ontology. Triple, a common way of representing relationships in a knowledge graph, is in the form of fact representing as $<Subject, Predicate, Object>$ where Subject belongs to the domain, Predicate belongs to relation set and Object belongs to the range of the relation. Two common instances of such triple in scholarly knowledge graph are $<Publication, cites, Publication>$ and $<Author, has\_paper, Institution>$ as shown in Fig. 2. Such homogeneous and heterogeneous relations respectively can be incorporated in the knowledge graph and enable it to link far-away entities in a meaningful and distinct way.

Lifecycle of knowledge graph incorporates various steps and tasks to perform A typical set of components are connected together to form *Scholarly knowledge graph construction workflow* from data representation to integration with applications as shown in Fig. 3:

- Semantically represented data model for scholarly communication: Data acquisition, designing of data structures for databases and domain ontology to represent the conceptualization of the domain are the preliminary step for knowledge graph construction. Data acquisition of the subject domain gains high importance due to the property of selecting representation of knowledge in scalable way. which contains entities (article, authors, venue) and relationships (cites, written_by) connecting those entities are included. Several labels are also connected which shows attributes and constraints associated with it. Designing the domain ontology to define classes and properties for unambiguous representation is an important task also. Annotation is the step in pipeline to annotate the content of scientific article with ontological concepts. However, acquiring the data from multiple resources and design of ontology from scratch are the challenging tasks as there many constraints have to be applied according to the subject matter.
- Information extraction, mapping extractions to an ontology and knowledge graph creation: Information extraction (IE) from scientific texts is a critical step in creating fine-grained scholarly knowledge graphs that characterize and connect scholarly articles. The intricate step of domain-specific and domain-independent information extraction requires extraction of scholarly entities and relationships. To follow an appropriate workflow, extracted knowledge is required to be mapped that reflects important rules and ontology patterns. Knowledge graph lifecycle starts with the process of extracting semantically correct annotated data and utilizing mappings to represent in a structured manner such as triplestore.
- Knowledge curation and quality assurance by domain experts: The rapid growth of scholarly metadata has been largely contributed by human which generates blatant errors. Measurement of quality (correctness and completeness) is accomplished by sampling with human moderators or crowdsourcing platforms. Knowledge graph curation process ensures the improvement in knowledge graph in terms of cleaning, organization, assessment and enrichment. Proposing an open and disjoint framework facilitates these tasks to be returned as a high-quality architecture over the heterogeneous resources. In addition to this, accurate communication of the properties and conditions to the mapping creator ensures flexible, reusable and significantly improved knowledge graphs.
- Management, deployment and application services:Automated semantic and syntactic integration of heterogeneous sources in knowledge graphs leads to managing machine understandable form and grant application developers to fabricate intelligent applications. Investigating search engines, ranked entities, recommendation services and question answering systems are remarkable applications powered by knowledge graphs.

### Common scholarly communication infrastructures

The scholarly communication community has derived numerous knowledge representation related projects listed in Table 2. Premium academic search engines exploits the scholarly data into knowledge graph structures that interlink the research entities through metadata. These infrastructures not only focuses on scholarly literature but also aims to generate a standardized system to gain linking between various artifacts such as authors, funders, projects, grants, venues, or source codes at semantic level. Common scholarly communication open graphs serves a number of purposes:

- The purpose of the scholarly infrastructure is to generate high coverage, high quality and uniform representation of the artifacts. For example, content of ResearchGraph [24] was originally provided in XML format, but was later made available to third-party applications using JSON-LD and Schema.org.
- The content of scholarly graph aims at integrating and aggregating various metadata records and supports analytics, monitoring, usage statistics, trends, discovery, recommendations and research impact assessment applications. For example, ORKG [25] enable features such as comparing research contributions [26], related work similarity [27] and automated extraction of information from literature [28]. Similarly, OAG [29] a heterogeneous entity graph is considered as benchmark for author name disambiguation, citation networks and comparing methodologies.
- Goal of scholarly infrastructures is to investigate applications for enrichment in order to promote discoverability of connections and investigation of artifacts, taking into account big data sets and linked data technologies. For example, ORKG incorporates scholarly literature integrated with data repositories to provide applications such as recommendations, reuse and visualization. In addition, OpenAIRE [30] connects trusted data sources to augment metadata and delivers value-added services such as mining, monitoring, and impact analysis. Infrastructures such as ORKG and OpenAIRE consider resources as fundamental entities to create the graph by employing the enrichment techniques. However, PID [31] instead of adapting this paradigm considers unique universal Persistent IDs itself from PID providers as the fundamental entity and create connections such as ORCID ID for researcher, Institution ID, and DOIs for metadata.
- The system’s usability and performance must be evaluated as part of the process of obtaining open source knowledge from various sources. To create high-quality data crowd-sourced comments, questionnaires, surveys, and comparative metrics are employed. Furthermore, the participation of open access platforms, repositories, and frameworks (services and software) such as DBLP, arXiv, and EasyChair is growing.
- All these endeavors are aimed at providing tools and services to assist research communities adapt Open Science publishing paradigms. For example, to make information retrieval easier, a faceted search system [32] is deployed to represent research contributions over ORKG. A number of open scholarly infrastructures do not offer services related to bibliographic data such as article citation and required to collaborate with bibliographic databases directly to support digital libraries. To encourage the community to create more realistic domain-specific infrastructures, a ready-to-use comprehensive benchmark data set as well as data injectors are needed. It is observed that most of the open scholarly graph investigates either the implicit or explicit representation and combining them in an unified knowledge graph remains a challenge. Furthermore, open scholarly graphs can push the boundaries of machine learning techniques and natural language processing approaches entirely, allowing for scalability and resilience.

**Table 2:** Graphs supporting scholarly infrastructures

Infrastructure	URL	Data representation format	Data size/no of triples	Data export	Ontology used	Linked data resources	Data access	Research entities
Microsoft Academic Knowledge Graph [33]	$$ ma-graph.org$$	RDF, N-Triple	Multidisciplinary, 210 million publications, 8 billion triples	SPARQL	Yes	MAG, DBpedia, Wikidata, OpenCitations, and the Global Research Identifier Database (GRID)	Open	Author, Paper, Citation, Field of study, Journal, Affiliation, Conference instance, Conference series
SciGraph [34, 35]	*scigraph*.*springernature*.*com*	JSON-LD, N-Triple, Turtle, RDF	2 billion triples	SPARQL	Yes	Springer Nature, Dimensions.ai, GRID	Open	Authors, Funders, grants, research projects, conferences, affiliations and publications
ScholarlyData [36]	*w*3*id*.*org*/*scholarlydata*	HTML, RDF-XML, N-Triples and JSON-LD	Computer Science conferences and workshops, 1,128,618 triples	SPARQL	Yes	Events, ORCID, DOI	Open	Academic event, Affiliation, Organization, person
OpenAIRE [30]	$develop.openaire.eu/graph-dumps.html$	RDF-XML, HTTP responses, RDF data, JSON	480Mi	SPARQL endpoint	–	Repository, Funders, Archives, databases, Publishers	Open	Literature, datasets, software, funders, grants, organizations, researchers, data sources
Open Research Knowledge Graph [25]	*orkg*.*org*	JSON, RDF serializations	–	REST API, SPARQL	Yes	Literature, Research repository and terminology	Open	Literature and its content
ResearchGraph [24]	*researchgraph*.*org*	XML, RDF-XML Triplestore, JSON-LD	250 million nodes	Cloud hosted services, REST API, GraphQL	Yes	PID, Literature, Repository, Publishers, Funders, aggregators, discovery	Controlled	Academic articles, datasets, funders, grants, organizations, researchers
OpenCitations [37]	*opencitations*.*net*/	RDF Triplestore	55M publications and 655M bibliographic citations	SPARQL	Yes	Bibliographic and citation metadata	Open	Researchers, Funders, Data repositories, Publishers
OpenResearch [38]	*openresearch*.*org*	CSV, RDF	Computer science Conferences, 9077 Events and 1061 Event series	ExportRDF, SPARQL	Yes	Repository, Funders, Archives, databases, Publishers, ORCID	Open	Events and its contents (EventTitle, country, topic)
PID [31]	*pidnotebooks*.*org*	RDF	30 million nodes	GraphQL	–	PID providers	Open	Publications, datasets, Software, Funders, Research Organization, Researcher
Open Academic Graph [29]	*www*.*openacademic*.*ai*/*oag*/	JSON	0.7 billion entities and 2 billion relationships	–	–	MAG and AMiner	Open	Venue, paper, Author, Affiliation

## Knowledge graph construction

Semantic richness and interlinked description of the content of scientific information has gained attraction over the last few years. By transforming scholarly document-centric workflows into knowledge graph information flows, the structure represents information semantically and express deep hidden interlinking among entities. The scholarly document-centric paradigm, on the other hand, has been critiqued for not allowing for automated knowledge processing, categorization, and reasoning. As a result, *Information extraction* (IE) of scientific entities and connections is required for organizing scientific information into structured knowledge bases. SKGs are scholarly knowledge graphs that incorporate metadata about research publications such as researchers, institutions, organizations, research subjects, and affiliations. However, various information extraction techniques are described in the literature to obtain fine-grained scholarly knowledge graphs. In order to automatically construct knowledge graphs, three categories such as domain-specific and domain-independent and cross-domain information extraction can be considered where input text and output format is crucial.

**Domain-specific IE** refers to extraction with the intuition that most scientific documents does not share common set of concepts and target specifically semantic depth of certain concept. This paradigm presents specific set of scientific concepts that can not generalize across various domains well.

**Domain-independent IE** paradigm presents a generic set of scientific concepts with no targeted information. The idea behind this extraction type is to extract all possible information structure present in the scientific document that is not normalized and canonical.

**Cross-domain IE** motivate to create relationshipsbetween entities across numerous domains with a high level of coverage, unless the structures are similar but the roles are different. Usage of external data sources such as DBpedia, which extracts information from Wikipedia is integrated in scholarly domain to create extended relationships and support cross-domain text classification tasks [39].

It is crucial to highlight that limited human supervision regarding the need for hand-crafted rules or human-labeled data set is required. However, manual intervention is still an essential step as it helps create gold standard data set generation for evaluation purposes. Aiming to fill this gap between knowledge exploitation ways in the defined domain, the general construction of KG has been customized to fit in various use-cases of the scholarly domain. The construction process incorporates top-down, bottom-up and mixed way of building knowledge graphs. The preset entity and relationship model graph may considerably improve the building quality and application efficiency of knowledge graphs in the scholarly domain. The knowledge graph construction can be classified into following categories based on the method used:

- First, studies that intended towards KG development utilizing machine learning techniques to leverage contextual data. Because the scholarly network has billions of nodes and edges, feature engineering and vector-based representation are becoming increasingly popular methods for processing raw data. For instance, techniques like deep neural networks and word2vec are employed to obtain precise syntactic analysis.
- Second, NLP techniques are widely employed since most strategies rely on the popular pre-trained language model and its modifications to do the extraction task. Technically, KG augmented by deep learning and NLP techniques better examines topological relationships and semantic meanings respectively, resulting in notable success in comprehending difficulties in scholarly domain and retrieving relevant solutions.
- Pattern-based acquisition methods are utilized to acquire the salient phrases from research contributions and attain phrasal granularity. The title of a scientific publications, for example, follows grammatical rules and includes scientific terminology at certain locations.

We focused and organized work here according to the order of approach used from machine learning approaches to NLP-based approaches and hybrid to rule-based approaches. This section summarizes the significant efforts involved in the direction of development/construction process scholarly knowledge graphs. The structure is simply logical, with the goal of maximizing the reasoning in our scenario.

### Information extraction

Information extraction of scientific documents is different from the traditional extraction methods as the understanding of full document is required compared to sentence level extraction. Concepts represent the implicit correlation and binary relationship from the perspective of conceptual hierarchy. The concept level hierarchical relationship is represented by entities and relationships, which are the extent and intent level objects, respectively. Named entities are used to represent general domains and KGs are constructed through entity and relation extraction often. However, subjects and objects also used to identify concepts and their attributes in scientific statements guided by the ontology.

**Entity/Relation extraction:** In [40], a unified multi-task learning model SCIIE is developed for entities recognition, relation extraction, and coreference clusters extraction. Six types for annotating scientific entities (Task, Method, Metric, Material, Other-ScientificTerm and Generic) and seven relation types (Compare, Part-of, Conjunction, Evaluate-for, Feature-of, Used-for, HyponymOf) is defined. A BERT-based model variant [41] is explored to identify relation types in knowledge graphs in scholarly domain. Farber in [42] developed a framework for extracting entities such as scientific methods and data set along with classification and aggregation. Similarly, several frameworks effectively revolve around the extraction of scientific metadata from scientific literature, SciREX (Dataset, Metric, Task, Method) [43], TDMSci (Tasks, Datasets and Evaluation Metrics) [44]. CORD-19 Named entities [45] are extracted and represent article’s title, abstract and body in RDF triplet format. In order to explore correlations with associated works rather than only its metadata, online scientific profiling [46] have been proposed to leverage the structure from scientific documents. CitationIE [47] is a domain-independent document level relation extraction. Another domain-independent NER method, CORD-NER [48] annotation based on pre-trained and guided supervised NER methods is implemented and tested on different data set. SciBERT [49] performed extensive experimentation on multi-domain corpus. Brack in [50] utilized abstracts of scholarly documents of ten different domains and annotated corpus is evaluated by human annotators. A cross-domain IE, for example PLUMBER [28] is presented comprising 33 reusable components and 264 different pipelines. The overall framework is trained over DBpedia and ORKG. Named entity extraction approaches, particularly those based on neural networks, require a large quantity of training data to get effective results. Because they neglect the context, the majority of IE systems are incapable of capturing the whole expression of a sentence.

**Concept level extraction:** To understand the structure and evolution of scientific fields, concepts are extracted from articles and represent scientific field as a knowledge graph. SciKGraph [51] proposed a framework to structure scientific field from the documents of that field by considering extracted concepts and keyphrases. Concepts are extracted and linked from Web of Science and Artificial Intelligence data using Babelnet graph-based approach and clustered on the basis of modularity. Similarly, an unsupervised model [52] is proposed to extract *is-a* and *ispropertyof* relations among entities using Part-of-speech (PoS) tagger. A taxonomy is constructed by combining the local taxonomies identified by the triples and further reduced to solve entity merging problem. The approach is compared to Open IE tools such as StanfordOpenIE and Reverb. It is important to note that, the evolution of the scientific field not only depends on the structure but also the concepts in common by calculating the similarity of the clusters. Same cluster represent same subarea and concepts are included or excluded from the subarea. Since most of the existing information extraction systems consider triples for reasoning in KG construction without considering specific property in scientific statements to compensate the limitation of flat representation of triples. In this view, [53] represents three layered SKG that extends BiLSTM model with MIMO sequence labeling approach to extract traditional triples as well as condition tuple for statement nodes. Proposed methods that extracts tuples outperformed as compared to existing OpenIE systems such as AllenNLP and Stanford OpenIE. In the context of structuring extra information instead of flat triple representation, a domain-independent Research Contribution Model (RCM) is proposed [54] that includes the schema of six core concepts by leveraging ontology.

**Table 3:** Information extraction from scientific documents

References	Extraction			Knowledge				
	Level	Input/field	Fact	Domain	Approach	Tasks	Source integration	Metrics
[40]	Entity	Abstracts	Triple	DI	Supervised	NER, RE, CR	–	P, R, F
[41]	Relation	Full-text	Triple	DS	Unsupervised	CLS	–	P, R, F, Accuracy
[42]	Entity	Abstract and full-text	Triple	DS	Conditional Random field	NER, CLS	SciBERT, MAKG	P, R, F
[43]	Entity and relation	Full-text	–	DI	Bi-LSTM	NER, CR, RE	SciBERT	P, R, F
[44]	Entity	Full-text	Triple	DS	Conditional Random field	SL	–	P, R, F
[45]	Entity	Full-text	Triple	DS	–	NER	DBpedia, Wikidata and BioPortal	-
[46]	Entity	Sentences	–	DS	Supervised	RE	–	P, R, F
[47]	Relation	Full-text	–	DI	TF-IDF, Graph embedding	CR, ECLS, RE	DBpedia, ORKG	P, R, F
[48]	Entity	Full-text	–	DI	Semi-supervised	NER	SciSpacy, UMLS	P, R, F
[49]	Entity	Full-text	–	DI	Unsupervised pre-training	NER, CLS, RCLS, Parsing	ScispaCy, BERT	F
[50]	Entity	Abstract	–	DI	Supervised	SL, CLS	–	P, R, F
[28]	Entity and relation	Sentences	Triple	CD	–	TE, CR, EL, RL	RoBERTa	P, R, F
[51]	Concept	Keyphrases	–	DS	Unsupervised	CLS	BabelNet	Accuracy
[52]	Concept	Sentence	Triples	DI	Unsupervised	RE	GROBID	P
[53]	Concept	Sentence	Tuple	DI	Semi-supervised	SL	–	P, R, F
[54]	Concept	Sentence	Quad	DS	–	–	–	–

Table 3 shows that the majority of work has been published on entity and relation level extraction. Input/Field represents the type of information considered for the extraction. A majority of studies have considered sentences from full-text of scientific articles rather than only abstract or title. There are only a few research on fact representation that have been published. However, there are only a few research that focus on extracting relationships between items from scholarly literature. In knowledge header, domain refers to the field of study that is selected to perform evaluation, e.g., Domain-specific (DS), Domain-independent (DI) and Cross-domain (CD). Approach refers to the algorithms applied on data and many authors have applied concepts of Conditional Random Field (CRF) in tasks such as NER, sequence labeling and classification. A set of NLP and Ml tasks are performed where NER, RE, CR, SL, TE, EL, RL and CLS refers to named entity resolution, relation extraction, coreference resolution, sequence labeling, triple extraction, entity linking, relation linking and classification respectively. Source integration refers to the vocabularies, language models, open scholarly infrastructures used to integrate and enrich the process of information extraction. P, R and F represents precision, recall and F-score respectively that have been calculated for majority of studies for evaluation. As far as concept level extraction is concerned, a handful studies are focused on extraction of phrases.

### Construction method level creation

Over the past few years, relevant techniques have been extensively used for the various applications such as scientific community analysis, clustering scientific fields and link prediction for research collaboration. Machine Learning and Artificial Intelligence have become the preferred methods for the processing and analysis of big data. Through semi-automatically extraction approach, the models are capable to collect and import entities captured from data sources.

**Fig. 4:**
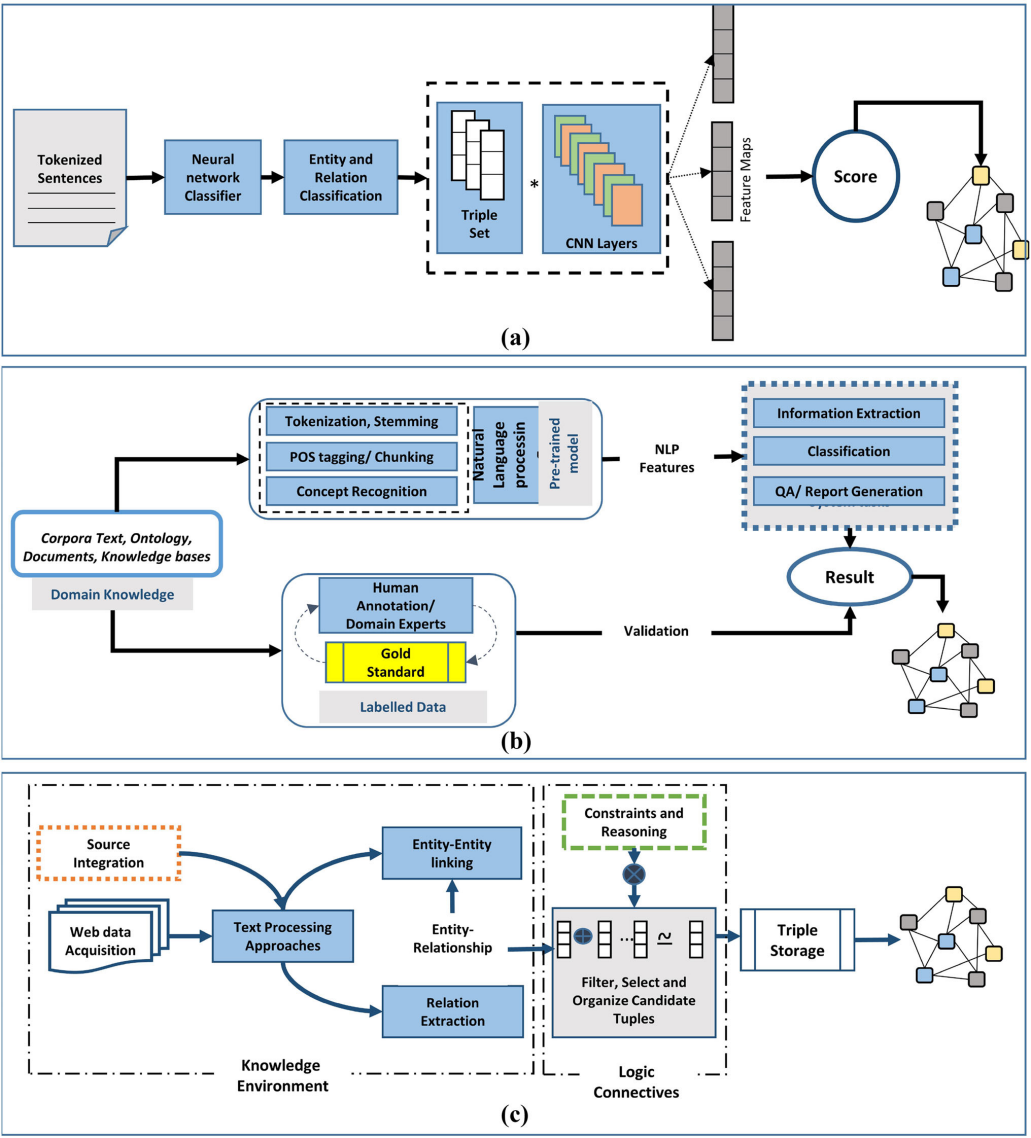
Algorithmic view of **a** neural network-enabled KG creation, **b** natural language processing-enabled KG creation, **c** rule learning-based knowledge graph creation

**Neural network-enabled KG creation** Various data-driven machine learning algorithms have been widely used in scholarly knowledge graph’s knowledge acquisition, construction and extracting critical information from vast data set. These approaches are used to solve the extraction level problems using word vectorization and feature extraction methods without considering the contextual information. On the other hand, these approaches have been used in generic automatic pipelines as well to construct knowledge graphs. Therefore, efforts of construction of knowledge graph using machine learning and deep learning algorithms are discussed as shown in Fig. 4a. For example, in the papers [55] and [56]document level extraction techniques are employed with graph learning techniques to explore text entity/relationship and summarization. A novel span-based mode [55]l, inspired by [40] is developed for entity and relationship classification by adding convolutional layers. This paper overcomes the disadvantage of imbalanced number of relations to increase the accuracy. Similarly, SCIERC is utilized to create summary knowledge graphs [56] using GAT model for node representation model extracted using DyGIE++. Quantitative analysis is performed using hand-crafted annotations and it is observed that unrelated relations are generated due to coreference resolution errors. These cascading issues are caused by the token-based approach’s fixed and sequential representation.

A fully automatic pipeline for knowledge graph creation for COVID-19 scientific literature incorporates applications such as literature discovery (research collaboration, article recommendation) and drug repurposing. A scientific knowledge graph [57] in former application category is constructed by considering structured and unstructured data from COVID-19 literature. Graph-of-docs and graph similarity measures are employed to generate features for link prediction task. A drug–drug interaction (DDI) prediction task is performed in [58] using KGE and a Conv-LSTM network is trained and analyzed. Fusion of various scientific sources is described including scientific literature and huge set of DDI triplet is constructed as RDF KG using semi-supervised technique. Another work ERLKG [59] utilized the COVID-19 literature by fine-tuning SciBERT for entity and relation extraction. The automatic pipeline of knowledge graph construction incorporates representation of entities and relationship into latent low dimensional space and fed into GCN-AE for link prediction task. In SoftwareKG [60], a bi-LSTM-based approach is used to generate a knowledge graph by identifying software mentions in scientific articles. Entity linking for disambiguation is performed using transfer learning methods.

Furthermore, bottom-up approaches are used to construct the knowledge graph using machine learning techniques in which, text mining and analytic is important step to implement. MatKG [61] framework is constructed using Naive Bayes Classifier to disambiguate authors. Similarly, statistical method is applied on geoscience literature to construct knowledge graph [62] in order to represent key facts in structured manner. Content words are segmented and represented using geology dictionary. Although majority of the approaches are based on supervised and unsupervised learning methods, it is worth noting that each submission seems to have its own methodology (techniques and phases) and seeks to achieve separate key goals in the knowledge graph construction process. In the literature, various algorithms of machine learning and deep learning approaches are combined with linguistic approaches for the information extraction as well as similarity measure tasks. This makes comparing approaches complicated, and a normalization of the learning process even more challenging.

**Natural language processing-enabled KG creation** Semantic enrichment entails the incorporation of metadata from scientific publications from many perspectives, as compared to the method that focuses exclusively on keywords or feature extraction. Scientific publications require automatic processing from human-readable format to machine-readable format. To understand the ability of model’s mechanism, extensively labeled data and pre-trained model is required. Though machine learning approaches outperformed human baselines in many specific cases but not properly integrates with prior knowledge of fine-tuned pre-trained data for interpreting the model’s behavior. Knowledge graph construction incorporates standard NLP tasks such as semantic role labeling, part-of-speech tagging, and chunking to get the best system along with pre-trained model as shown in Fig. 4b. A pipeline for literature-based biomedical knowledge graph [63] is proposed to extract biomedical entities and integrate it with prediction methods on Parkinson’s disease. Entities and relationships are extracted using SemRep NLP program and evaluated manually to observe misleading entities.

In [64, 65] KGen is presented to develop knowledge graphs from abstract of scientific documents by extracting triples using Semantic Role Labeling (SRL) and PoS tagger. However, KGen employed tools to design automatic methodology still human intervention is the requirement of the technique to manually update and review intermediate results. Limitations such as lack of SPARQL endpoint and inclusion of side information are improved in by mapping UMLS and generating secondary set of triplets respectively. In [66], metadata is trained and passed to two layered bi-directional LSTM to accomplish entity extraction task. Similarly, a scalable, semi-supervised and domain-independent method [67] is proposed for extracting concepts from scientific literature using word embedding and pre-trained BERT model. To avoid misinformation in resources and to generate reliable knowledge graph for drug repurposing, COVID-KG [68] is constructed using hierarchical spherical embedding and text embedding in the direction considering cross-media (text and figures) extraction. Proposed KG is evaluated retrospectively by domain experts for coarse-grained and fine-grained entity, relation and event extraction.

**Rule learning-based knowledge graph creation** To express links and dependencies between entities in datasets and to capture the underlying patterns in data, rules are commonly utilized. Rules plays crucial role in automated reasoning and finding inferences. A mainstream technique in rule-based reasoning is to formalize the problem and to obtain the inferences as per predefined rules. Second, rules can be predicted by applying statistical reasoning approach directly to filter, select and organize candidate tuples as shown in Fig. 4c. There are some efforts where the advantages of both techniques are combined and presented the final form of reasoning to achieve the goal of completing multiple tasks. A literature knowledge graph [69] is proposed where abstract is represented as the decomposition into four sub-domains (Background, Objectives, Solutions, Findings). To avoid the labor extensive task of manual ontology element identification, an automatic ontology element identification is proposed using text classification based on semantics. The input sentence is translated into embedding vectors and output vector is utilized for classification. Patterns of abstract structure are identified and high precision is obtained for identification and classification of abstract in four sub-domains. On the surface, knowledge discovery via reasoning over the embedding appears to convey knowledge in a coherent framework. It covers significant areas of the literature and also improves the quality of learnt rules. A heterogeneous SCM-KG scholarly communication metadata-knowledge graph [70] is presented in which SWRC and FOAF ontologies are reused to create core vocabulary. In this paper, distributed schemas (DBLP and MAG) are integrated and parallelization in rule-based data mappings is implemented. The use of semantic similarity measures in conjunction with RDF interlinking to assess the relatedness of concepts in two resources is demonstrated. Assessment of proposed pipeline is evaluated on the parameters of completeness, accuracy and execution times of query processing per second in the linking step.

**Table 4:** Scholarly knowledge graph construction

References	Extraction					Knowledge					
	Level	Input/field	Fact	Entity	Relation	Domain	Approach	Tasks	Source/tool integration	Metrics	Application
[55]	ER	Text	Span	Task, Method, Metric, Material, Other-ScientificTerm and Generic	Compare, Part-of, Conjunction, Feature-of, Used-for, HyponymOf	DI	NN Classifier	Classification	SciBERT, JSON	P, R, F	–
[56]	ER	Text	Triple	Task, Method, Metric, Material, Other-ScientificTerm and Generic	Compare, Part-of, Conjunction, Evaluate-for, Feature-of, Used-for, HyponymOf	DI	GAT	Entity Alignment and Deduplication	DyGIE++	P, R, F	Summary generation
[57]	ER	Text	-	Paper, Word, Author, Laboratory, location, Institution	Cites, is_similar, includes, connects, writes, co_authors, affiliates_with	DS	Word Embedding	Link prediction	CSV, Neo4j	Accuracy, R, P	Discovering future research collaborations
[58]	ER	Text	Triple	Drugs, genes, proteins, pathways and enzymes	HasTarget, hasEnzyme, hasTransporter, isPresentIn, isImplicatedIn	DS	CNN and LSTM network	Classification	Bio2RDF, SPARQL	P, R, F	Drug–drug interaction prediction
[59]	ER	Text	Triple	CHEMICAL, PROTEIN, DISEASE	CHEMICAL-PROTEIN, CHEMICAL-INDUCED-DISEASE	DS	Graph Convolution Network Auto Encoder	Link Prediction	SciBERT	P	Association of biomedical entities
[60]	Concept	Text	Triple	Software mentions	Replaced_by	DS	Bi-LSTM, transfer learning	Entity disambiguation	JSON-LD, SPARQL	Manual, F	Software usage in social science
[61]	Concept	Text	–	Author, Material	–	DS	Naive Bayes Classifier, CTANE	Classification, Deduplication		P, R	Scientific research trend analysis
[62]	Concepts	Text	–	–	–	DS	Conditional random Field, TF-IDF	Content segmentation and extraction	–	P, R, F	Chinese word extraction from Geoscience literature
[63]	ER	Text	Triple	Disease, Patient	Treat, Not treat	DS	SemRep	Classification	–	P, F	Drug Repurposing
[64, 65]	ER	Text	Triple	–	Agent, Patient	DI	Semantic Role Labeling	Ontology Linking	Stanford’s CoreNLP, RDF turtle	Manual	Semi-automatic method to generate KG
[67]	Concepts	Text	–	Title, Abstract and Citation	Cited, Aim, Method, Result	DI	Sequence labeling, BERT embeddings	Concept Extraction, Graph Construction	DBSCAN	P, R, F	Research trend analysis
[68]	E	Text and figures	Triple	Gene nodes, Disease nodes, Chemical nodes, and Organism	Gene-Chemical-Interaction Relationships, Chemical-Disease Associations, Gene-Disease Associations, Chemical-GO Enrichment Associations and Chemical-Pathway Enrichment Associations	DI	Sequence embedding	NER, Event extraction	OCR, BioBert	Manual, F	Multimedia extraction, Question answering, report generation
[66]	Concept	Text	Keyphrases	Papers, Authors,entities, entities mentions	Citations, Authorship, mention-mention, Entity-entity relations	DI	Sequence labeling	Entity extraction, Linking	Tagme, MetaMap Lite, ScienceParse	P, R	Data discovery and ranking
[69]	Concepts	Text	–	Background, objective, solution, and finding	–	DI	BERT	Reasoning	BERT, CSV, SPARQL	P, R	Abstract Knowledge representation and ontology element identification
[70]	ER	Text	Triple	Paper, Author, Affiliations	–	CD	Rule mapping	Instance matching	MAG, DBLP, SWRC ontology, Dublin Core and FOAF, Scrapy, CSV, SPARQL	R, Accuracy	To create KG pipeline

Table 4 presents the studies that incorporated neural network-based, NLP-based and rule-based approaches. There are research that define the domain semi-automatically in order to retrieve a subset of manually defined types. Studies, on the other hand, have used approaches to find new types from unlabeled data. It is easy to derive entity and relations from the corpus using these semi-automatic approaches. However, these methods provide extractions with a modest level of precision and noise. Furthermore, fusion level development using entirely off-the-shelf OpenIE techniques as well as ontologies that are built or reused is covered.

### Knowledge fusion level creation

Semantic web technologies, which describe domain knowledge using diverse concepts such as ontologies, Open Information extraction (OpenIE) tools and query processing languages, enable the display of domain information in machine-readable ways. The goal of knowledge integration is to create ontology and taxonomy to represent hierarchical structure. Knowledge fusion helps in generating metadata from various data sources as well. Use of common ontologies and general metadata from schema.org is required to ensure the quality of the knowledge graph. In addition, Knowledge graph have been facilitated with open extraction tools such as OpenIE to feature the knowledge resources.

**Schema based:** As we are transitioning from big data to semantic data, KGs play an important role as critical component of semantic web. Since knowledge graphs have emerged as an technology with broad application areas, it seeks integration with standard third-party resources such as ontology and vocabularies. In this context, author presented property graph [71] where RDF generation, annotation and knowledge graph in agriculture domain is populated by adding domain knowledge. Properties of a set of ontologies is reused to convert scientific articles into RDF format. KG-COVID-19 [72] a fusion-based KG, incorporates the design principles such as reproducibility, interoperability and provenance to provide flexibility and quality by leveraging modern ontology best practices. Framework is divided into fetching data, converting into KGX format and merging steps by preserving properties. It supports ontology-enabled data sources for drug repurposing and Biolink model to categorize nodes and edges qualifying for ingestion from multiple sources. Further, the model is embedded, trained and tested for machine learning applications and visualized using t-SNE plot. A RDF graph-based on ocean science named OceanGraph [73] is proposed that reuse vocabularies and ontologies over the domain of biodiversity. OpenBiodiv [74] is the biodiversity knowledge graph based on FAIR-Linked data that utilized scholarly publishing and biodiversity-specific ontologies for conceptual modeling. These current approaches utilizing existing ontologies and vocabularies to annotate the context at long text level that are semantically far from each other.

Academia/Industry DynAmics (AIDA) Knowledge Graph [75] is introduced and generated by integration of MAG, Dimensions, English DBpedia, CSO and GRID. AIDA knowledge graph describes 21M papers and patents according to the research topics drawn from CSO. In this paper, the relationship between industry and academia is analyzed due to unremitting engagements by exploiting the corpora of research articles and patents. A knowledge-driven framework KORONA [76], is presented to unveil the scholarly communities for the prediction of scholarly networks. To generate KG, development stage uses mapping rules between the Korona ontology that utilizes the homophily prediction principle and the incoming data sources. These applications are limited to the expert’s domain, and because the expert knowledge base is heavily reliant on experts’ experiences, it is difficult to transform it across domains.

**Off-the-Shelf tools based:** In general, NLP tasks such as document summarization, fact verification and retrieval requires to take huge data and pruning need to be perform over different document contexts. Various studies handle these tasks with the help of OpenIE tools where each KG is generated. In this context, a literature knowledge graph for clinical research methodology dataset OIE4KGC [77] is generated using the concept of open information extraction. In this paper, spacy’s Noun chunker is used to retrain noun phrases and filtered triple such as$$< study, determine, cardiovascular risk factors>$$.Finally, concept and document vertices are linked having “mentions” and edges link a pair of concepts denote relations extracted using OIE. Furthermore, in [78] implements the Stanford Core NLP PoS tagger, which extracts predicate between the entities recognized by the Extractor Framework and the CSO Classifier via the PoS Tagger.

In order to generate the knowledge graph, issues such as multiple entities refer to same concept, redundant relationships and generic entities are addressed. A scientific knowledge graph [79] is presented that analyses research publications in the field of semantic web using a set of NLP and Deep Learning approaches. Entities and relations are extracted from literature using extractor tool [40] and discarded generic relations. CSO classifier is used to automatically classify research articles conforming to Computer Science Ontology [80]. Further, the output is processed with OpenIE to retrieve all set of triples. To remove multiple entity issue during graph generation phase entity merging task exploits Levenshtein similarity technique considering that relation merging task exploited Word2Vec word embeddings and cluster algorithms. Two main challenges such as disambiguation of entities and specificity of relations are addressed in this paper. In [81], artificial intelligence knowledge graph (AI-KG) is presented that includes 820K research entities, 14M RDF triples from 333K research publications in the field of AI. AI-KG used DyGIE++, Stanford CoreNLP and the CSO Classifier that extracts entities and relationships. It uses BERT embeddings based framework to analyze scientific text and then CSO classifier and OpenIE are applied for parsing. It filters the resulting entities and removed entities that were not present in the CSO topics list. It integrated to map all three subsets of triples using Word2Vec (Titles and abstract) and semantic technologies such as silhouette-width measure in order to quantify and qualify as valid triple. In this approach a MLP classifier is also used to move the triple from invalid set to valid set of triples in order to refine the set of consistent triplets. Another work in this direction CKG [82] is presented by extracting rich information by considering semantic (SciBERT) as well as topological information (TransE). Normalization and linking techniques are applied to eliminate noisy author and citation concepts by thresholding confidence score. CKG is used as article recommendation as well as information retrieval to search author leaders, institutional leaders and collaborations.

Ontologies are essential aspects of academic knowledge networks that conceptualize scientific semantic communication. The description of various concepts and objects, as well as their relationships, is used in the formation and understanding of ontology. The majority of work has considered several domain-specific ontologies and supplemented the data sources by providing patterns with unique instances, as seen in Table 5. The usage of ontology assumes expert input, which leads to bias behavior in favor of precision and increases the cost. Open domain IE, on the other hand, has been used to treat any noun phrase as a candidate entity and any verb phrase as a relation candidate. In general, tagging and parsing are used to extract features, and then classifiers are used to produce a score. The fundamental benefit of using openIE paradigms is that they can be simply applied to big corpora with no need for training data. Off-the-shelf techniques can be used to extract data from new scholarly data sources in this scenario wi. However, it is unable to distinguish different surface forms for the same object or relation, resulting in poor aggregation performance.

**Table 5:** Scholarly knowledge graph fusion-based construction

References	Extraction					Knowledge				
	Level	Input/field	Fact	Entity	Relation	Domain	Tasks	Source Integration	Metrics	Application
[71]	Keyphrases	Text	Triple	–	–	DS	Annotation	RDFization	Bibo, foaf, prov, Wikidata, sio, RDF, Neo4j	Scientific literature semantic data management in agriculture domain
[72]	Concept	Text	Triple	Publication, OntologyClass, Drug, ChemicalSubstance, BiologicalProcess, Disease, Protein, Gene, PhenotypicFeature, MolecularActivity	–	DS	Classification, link prediction	Biolink, HPO and the Mondo disease ontology, RO, RDF, Neo4j	–	Prediction and querying
[73]	ER	Text	Triple	Publications, people, Campaigns, Environmental variables, Species, locations	Contributor, has_subject, reported_by, participant, has_measurement, collect, recorded_by, has_place	DS	Cross linking	NMDS, GBIF, OBIS, foaf, RDF, SPARQL	–	Data augmentation and meta analysis for ocean science
[74]	E	Text	Triple	Article, Title, DOI, Introduction, Author Name, Treatment, Nomenclature, Materials, section, Taxonomy concepts	–	DS	Disambiguation	SPAR, foaf, RDF4R and ROpenBio, RDF, SPARQL	Competency questions	FAIR-complaint biodiversity literature-based knowledge management system
[75]	ER	Text	Triple	Publications, patents, topics and industrial sectors	HasTopic, hasAffiliationType, hasAssigneeType, hasIndustrialSector	CD	Topic detection, Classification	DBpedia, MAG, CSO, GRID, SKO, PROV-O, INDUSO	Manual	Cross-domain knowledge graph
[76]	ER	Text	Triple	Publications, researchers, publication venues, scientific institutions	Co-authorship, citation, and collaboration	DS	Network detection	METIS, SemEP, KORONA ontology		To generate communities of researchers for Collaboration recommendation based on Co-author networks
[77]	Concepts	Text	Triple	Concepts and documents	Mentions	DI	Triple filtering, linking concepts	RnnOIE, RDF, Cypher	P, R, F	Open information extraction and literature KG from clinical trials methodological articles
[78]	ER	Text	Triple	Task, Method, Metric, Material, Other-ScientificTerm and Generic	Compare, Part-of, Conjunction, Evaluate-for, Feature-of, Used-for, HyponymOf	DI	Triple refining and Entity merging	OpenIE, CSO classifier	P, R, F, Manual	Generic knowledge graph construction
[81]	ER	Text	Triples	Research topics, tasks, methods, metrics, materials	Verbs (uses, includes, is, evaluates, provides, supports, improves, requires, and predicts)	DS	ER extraction	DyGIE++, Stanford CoreNLP, CSO Classifier	P, R, F	Domain-specific KG generation
[79]	ER	Text	Triple	Task, Method, Metric, Material, Other-ScientificTerm and Generic	Compare, Part-of, Conjunction, Evaluate-for, Feature-of, Used-for, HyponymOf	DS	Entity relationship merging	OpenIE, CSO classifier	Manual	KG construction using openIE
[82]	E	Text	Triple	Paper, Authors, Institution, Concepts, Topics	Authored_by, affiliated_with, associated_concept, associated_topic, cites	DS	Concept,author normalization, Citation linking	Comprehend Medical, Apache TinkerPop Gremlin and SPARQL	Manual	Question answering and paper recommendations

### Discussion

Despite the promise and benefits of harnessing knowledge graphs for scholarly communication, we are still in the early stages of development, with many unanswered problems. (a) How can we incorporate more specialized scientists in the curation process? (b) Do the semantic curation strategies scale across vast topic areas and semantic representation be achieved? (c) How varied structured data models can contribute to give meaningful path for knowledge graph? Typically two types of directions have been used in the literature to populate the knowledge graph either by human experts or by applying linguistics techniques and machine learning approaches. With a few exceptions, these studies rely on the manual effort of annotation which requires experts to extract background knowledge. In addition, an article leads to high number of entities when full-text is considered for annotation. The domain-specific extraction process requires domain experts and annotators, which makes the extraction process costlier and limited. However, domain-independent KGs are generic within-sentence extraction. The first way to populate knowledge graph generates high-quality and validated outcomes with improved precision-recall analysis. However, it suffers from limited scalability issue as well as manual effort consuming. In comparison, the latter produces nosier outcomes but can handle huge corpora of scientific documents. To keep the human out of the construction of knowledge graphs, an automatic pipeline integrating IE and KG creation is the most vital step for the structured or unstructured metadata.

A wide range of studies using natural language processing techniques can be found that applied over a collection of scientific articles. For speeding up the extraction process in scientific publications, a collection of natural language processing algorithms supporting OpenIE and ontologies is used to generate an end-to-end automatic pipeline for the generation of knowledge graphs. It is worth mentioning that most of the studies in this section analyzed natural language using basic extraction, mapping, tagging, and parsing technologies. Second, several domain-specific ontologies have been widely employed to cover all of the data in various sections of the study.

A rule-based approach gathers key scholarly information in the form of patterns, leveraging regular expressions in title, abstract, research problems, application areas, and citation information. However, the knowledge graph’s reasoning capacity is reduced by its insufficient integration of subjective information from the literature. Citation data, for example, is useful for quantifying bibliometric and trend analysis but offers less information about the content of the paper. As a result, the mapping rules should be tailored to the distinct types and formats of data sources and trained accordingly. Furthermore, manually curated rule generation and mappings result in gold standard data, however this curation can be skewed toward certain well-known issues and limited to the expert domain. Hybrid reasoning, which incorporates the use of ontologies, knowledge completion methods, and schema construction, is critical and improves performance of KG creation.

In addition, very few studies are using extractor framework such as OpenIE, DyGIE++ and RnnOIE to automatically extract the entities. As a result of using extractor framework, a huge number of entities and relationships referring to the same concept is detected. However, the extracted information is too generic and require further normalization to remove overlapping and redundancy of extracted information. Moreover, evaluation and standardization becomes difficult during application on larger scale due to domain-independence and misclassification. Besides, consideration of coreference resolution is also being ignored till date during information extraction and its applications. Resolving syntax complexities and elimination of ambiguated text are also the part of the NLP extraction pipelines. [40] have extracted coreference links using shared span representation and avoided cascading errors. We discovered that entity coreference issues have a significant influence on predicted graphs, and that our models need to make it simpler to capture these flaws in interactions. Fusion of visual semantics and textual semantics have gradually emerged as new direction in knowledge graphs also. In the literature [68] that creates multi-modal KG using derived knowledge from graphics and diagrams in addition to plain text except. For example, generation of multi-modal KG provides better query experience in applications by extending concept set. In addition, studies lack coverage for important entity types (e.g., affiliations) and domains (e.g., physics).

## Knowledge graph utilization in scholarly domain

The utilization of knowledge graph refers to the communication with stakeholders as well as usage of the already build KG as input in scholarly domain. Need for interactive front-ends and querying endpoints is still essential to view insightful results. It includes flexible access methods, import/export result formats, visualizations such as dashboards and leaderboards for the user-friendly interactions. The utmost relevance of this phase is to analyze the usage of knowledge graphs as input and tools, system interfaces as output on the top of the database supported by the knowledge graphs. In this section, some efforts in this directions have been discussed that generates natural language descriptions and visualization of results.

To generate natural language descriptions from KG, GraphWriter, a graph encoding–decoding model is performed by building on Graph attention network [86]. A novel Abstract GENeration DAtaset (AGENDA) is created from Semantic Scholar corpus [66] to generate an abstract automatically. During encoding step, publication title and knowledge graph are encoded by computing hidden representations using GAT for each node. During decoding step, vocabulary and copy mechanism from knowledge graph is utilized to generate sentences. It is shown that proposed approach utilizes the power of knowledge graphs along with title of publication and generates largest gain. Graformer [83], which used encoder-decoder architecture on the AGENDA data set to interpret shortest path and learn about graph structure to depict related global and local pattern information, is another contribution in this direction. These researches have been included in this section because generating a natural language description from KG makes the stored information more accessible to a wider group of end users in terms of question responding and interpretability. In order to support knowledge provenance, Whyis [84] a biology KG is constructed using nanopublications and deployed as assertion graph to represent drug–protein–disease interactions demo is presented by analyzing the probability of inlinks and outlinks of the node.

A crowdsourcing enabled initiative to convert document oriented information flow to knowledge-based is pre- sented to generate research domain overview to write survey articles. Aurora [90] is proposed that exploits semantic representation of OpenResearch.org. CL-scholar [88] that utilized meta path to represent semantic relations and OCR++ framework is used for textual and network information extraction task. Further, ranking based on popularity is employed and deployed. Similarly, a cause–effect knowledge graph [89] is constructed and represented by a web application for better exploration and querying. It utilized biological expression language (BEL) scripts and developed using biological knowledge miner (BiKMi) for drug repurposing.

Further, [90] uses existing SciKGraph framework to construct knowledge graph and proposed a visualization tool to get researchers connected with the evolution of scientific concepts. An application of AIDA KG, ResearchFlow [91] which forecasts and quantifies the influence of research topics on industry. It analyzed that 89.8% topics first evolved in academia and then preceded by industrial research publications and patents. In addition, AIDA dashboard [92] is also developed to represent statistical analysis such as citation analysis, conference similarity and trendy topics by leveraging AIDA knowledge graph. In addition, TDMS-IE [93] is developed for an automatic identification of tasks, datasets, evaluation metrics (TDM) triples to extract resultant best numeric score from scientific papers of NLP domain. Most importantly, key difference is that entire paper instead of only abstract is analyzed for the construction of the leaderboard containing TDM. Leaderboard is the form of meta analysis summary that provides appropriate literature for comparisons of proposed methods as well as selection of baselines to compare against. Document and table score representation is defined followed by paper tagging from the taxonomy and two datasets are created to test the proposed system. For further improved semantic visualization task, Kibana dashboard is created to show global view of process–disease relations through heatmap in [68]. The basic structure of a large knowledge graph can be easily shown with a limited perspective, but portraying cross-linked sources and exploratory tasks is cumbersome. SemSpect [94] is a client server application that explores answers from RDF graphs and depicts group of objects using predetermined classification techniques.

In order to visualize and explore the information from CORD-19 data set, [45, 89, 95] integrated with data transformation, entity linking and analytic tools. [45] integrated platforms such as Corese and MGExplorer. A Covid Linked Data Visualizer is developed to view node edge, clustering based and egocentric visualizations. Several Jupyter and R notebooks are designed in the form of dataframes to represent query results related to co-occurrences of the diseases in the articles. A Knowledge graph toolkit (KGTK) [95] is proposed to harness the capabilities of knowledge graphs to manipulate, retrieve and analysis in real-world scenarios. It supports importing/ exporting, filtering, embedding and graph statistics data science operations.

Few papers focus majorly on operation for retrieving and manipulations, on the other hand rest focus on storage and visualization as shown in Table 6. Graph processing capacity and computational powers of graph databases is utilized with the help of graph structure. GraphDB is highly efficient in storing and accessing graph database and allows exploring RDF classes to access instances. On the other hand, a number of studies used Neo4j for data storage, querying and visualization considerably as compared to the native triple storage platforms. As Neo4j query language named Cypher is easy to use as compared to GRAPHQL and various plugins are also available to extend its functionality.

**Table 6:** Scholarly knowledge graphs utilization

Model	Objective	Key features	Link for visualization	Data model/domain	Method used	Technical details
GraphWriter	KG Utilization	Graph to text generation	–	AGENDA	GAT capturing global context	–
Graformer [83]	KG Utilization	Graph to text generation	–	AGENDA and WebNLG	Self-attention Graph method	–
SciKGraph [90]	Visualization	Tracks the evolution of a scientific field at a concept level	*github*.*com*/*maurodlt*/*SciKGraph*	SciKGraph framework	Clustering	Python 3.7, flask 1.1.1, HTML 5,CSS 3, Bootstrap 3.3.7, and javascript 6
ResearchFlow [91]	KG Utilization	To quantify the research topic trends across academia and industry	*w*3*id*.*ord*/*aida*	AIDA KG	Diachronic analysis	–
AIDA dashboard [92]	Visualization, Web application	Analytics about research dynamics	*w*3*id*.*org*/*aida*/*dashboard*	AIDA KG	Classification and tagging	Python, HTML5 and Javascript
Aurora [90]	Querying	Generates overviews of research domains	$openresearch.org/wiki/Papers_query1$	OpenResearch	Crowdsourcing platform	SPARQL endpoint
TDMS-IE [93]	Tabular visualization	Automatic construction of NLP Leaderboard and summarize scientific results	$github.com/IBM/science-result-extractor$	NLP-TDMS	Classification	–
CL-scholar [88]	Querying	Search and explores current research progress in the computational linguistics community	*cnerg*.*iitkgp*.*ac*.*in*/*aclakg*	ACL Anthology	OCR++ for extracting metadata	ReactJS, supports REST API, NodeJS server, MongoDB
Whyis [84]	KG Exploration	Semantic meta analysis capabilities	$bit.ly/whyis-demo$	DrugBank, Uniprot	Stouffer’s Z-Method	Extensible Stylesheets Language Template (XSLT) to generate RDF
Covid-KG [68]	Visualization	Dense tag clouds and heatmaps	*github*.*com*/*elastic*/*kibana*	CORD-19	Data indexing	Elasticsearch and Kibana dashboard
SemSpect [94]	Visualization	Aggregated Tree overview	*scigraph*.*semspect*.*de*	SciGraph	Classification	Cient-server Application HTML5/JavaScript UI, Java REST backend, Neo4j for storage
Covid Linked Data Visualizer [45]	Visualization and querying	Enriching, reusing and adapting pipeline	*covid*19.*i*3*s*.*unice*.*fr* : 8080	CORD-19	Argumentative Clinical Trial Analysis tool	Python and R Jupyter notebooks, JSON format, SPARQL endpoint
BiKMi [89]	Web Application	Cause-and-effect network	$bikmi.covid19-knowledgespace.de$	CORD-19	Biological Expression Language derived network	Python Django and OrientDB
KGTK [95]	KG Utilization and exploration	Represents graphs in tables for data science applications	$github.com/usc-isi-i2/kgtk/$	CORD-19	ConceptNet, BERT	Scikit-learn, SpaCy, TSV for edges, RDF, Neo4j, Gephi, SPARQL

## Knowledge graph refinement

A series of studies argued that many state-of-the-art methods do not consider the semantic distance among the entities and relations. Knowledge graph embedding [96] is the representation of the entities and relations among entities in a continuous vector space. This representation then further models the interaction among entities to solve knowledge completion task. The knowledge graph embedding models a triple of the form $$<Head, relation, Tail>$$ as input, computes matching score and predict the validity of each triplet. The embedding vectors contain rich information about entities and relationships and learned embeddings can be used in tasks such as entity classification and link prediction/ knowledge graph completion [97]. Link prediction aims to predict missing relations, while classifying entities aims to define classes of different entities. In general, knowledge graph embedding model can be categorizes such as translation-distance-based model, neural network-based model and multiplicative model. Following terms are required to understand the approaches: *Score Function:* The score function takes a triple’s embedding vectors (*h*, *r*, *t*) and produces a value that indicates whether the triple is a fact or not. A triple’s score should be greater if it is more plausible. *Negative sampling:* For a triple (*h*, *r*, *t*),  a negative sample is formed by replacing either h or t with a random entity $$(h'$$ or $$t')$$ from set of entities. *Loss function:* Initially, positive and negative triple scores are created at random, and the loss function is optimized so that positive triples get higher scores than negative triples. In this section we focus on various types of embedding methods and the applications scenarios of embedding vectors in scholarly domain-specific knowledge graphs.

### Translation-based models

Translation-based approach is one of the most common KG embedding model where each entity is modeled as point in vector space and each relation is modeled as an translation operation. This approach maps the head entity and relation to be close to the embedding of the tail entity by minimizing the score of the triple. Subsequently, various models have been proposed that improves the capability of the basic translation models.

An improvement in existing translation model, Trans4E [98] is designed to remove the issue of relationship cardinality such as *(hasTopic)* where, head entity (*h*) is very high in number as compared to the tail entity (*t*). Such conditions costs computationally high and unable to distinguish well among embedding vector which is handled by applying transformations. Similarly, in [99] authors have applied various translational methods and TransD outperformed in the constructed heterogeneous bibliographic network. TransD creates mapping matrices based on entities and relations, in order to capture the heterogeneity of both entities and relationships at the same time. In this paper, authors found TransD to be better model instead of others due to its benefit of using two vectors to represent each entity and relationships.

Another co-authorship link prediction task on scholarly Knowledge graphs [100] is proposed with soft margin loss function. Exploration of many to many co-authorship relations is the objective of providing predicted links. This study shows the robustness of the model using TransE-SM loss function to deal with undesirable effects of false negative samples. Instead of using margin ranking loss, the optimization utilizes slack variable $\xi _{h,t}^r$ to alleviate the negative effect of the generated negative samples and $(\gamma _2-\gamma _1)$ is the margin. The score function is defined as $f_r{(h,t)}$ where $S^-$ and $S^+$ are negative and positive sample sets.

$$\begin{aligned}&\min _{\xi {^r_{h,t}}} {\sum _{(h,r,t)\in S^+} {\xi _{h,t}^r}^2}\\&f_r{(h,t)} \le \gamma _1, \quad (h,r,t) \in S^+\\&f_r{(h',t')} \ge \gamma _2-{\xi _{h,t}^r}, \quad (h',r,t') \in S^-. \end{aligned}$$

It is observed that the embedding vectors are semantically far from original mappings and generate ambiguous entity pairs in translation-based models. To make vectors semantically close TransP [101], a novel translation with penalty-based embedding model is taken into consideration. A novel Relation Embedding method based on local context is proposed to enhance the entity typing performance followed by keyword extraction method to highlight critical concepts in selective bibliographies. Scoring function $f_v(h,t )$ is the distance between $h+v$ and *t* whereas loss function $\mathcal {L}$ where $\gamma$ is the margin encouraging the difference between true triples and false ones.

$$\begin{aligned}&f_v(h,t ) =\Vert h+v-t\Vert ^2_2+ \lambda _1\Vert h-h_c\Vert _2^2+\lambda _2\Vert t-t_c\Vert _2^2\\&{\mathcal {L}}=\sum _{(h,v,t)\in G}\sum _{(h',v',t')\in G'}[\gamma +f_v(h.t)-f_v'(h',t')]_+. \end{aligned}$$

In order to analyze text embedding along with graph embedding techniques, an entity retrieval prototype [102] is presented which utilizes both textual information and structure information. A novel co-author inference evaluation is carried out to show the effectiveness of the TransE knowledge graph embedding models for entity retrieval. However, TransE have not shown significant improvement alone due to sparsity issue of the entity such as Paper. Similarly, [103] proposed generic literature-based knowledge graph approach to predict drugs that extracted triple using SemRep tool and further filtering is applied using knowledge representation learning methods. It is important to note that during filtering unnecessary relations were removed and normalized on the basis of degree and score assigned. However, TransE outperformed over all KRL applied. To overcome the problem of opaque predictions, discovery patterns were explored intuitively over five new drugs to obtain potential specific explanations such as (drug INHIBITS gene CAUSES COVID-19), (drug INTERACTS_WITH gene PREDISPOSES COVID-19) etc. Scholarly communication domain is conceptualized to create a knowledge graph for metaresearch recommendations (SG4MR) [104] as link prediction task. Created knowledge graph is tested on translational as well as Description-Embodied Knowledge Representation Learning models. The aim is to capture textual information well by applying textual and structural embedding but TransE outperformed over the description-based representations. Another work in this direction is proposed as Cov-KGE [105] that utilized low vector space on large corpora Pubmed using RotatE. Further, enrichment analysis of gene set is performed to validate the predictions on various data sets. In order to minimize distance between negative and positive links loss function is utilized:

$$\begin{aligned} L=-\log \sigma (\gamma -d_r(h,t))-\sum _{i=1}p{(h_i,r,t_i)}. \end{aligned}$$

An improvement in [63] is employed by integrating the existing medical knowledge graph with KG completion methods such as TransE and TransH to consider all interactions. TransH outperformed TransE due to its reasonable behavior in different relational hyperplanes and TransE’s shortcomings in handling cardinality. In another paper [59], TransD is the best performing entity representation learning method for link prediction task. To capture the diversity of chemical-protein or chemical-disease type entities, the project matrices are determined by both entities and relations. Hierarchical relationships, which are particularly prevalent in knowledge graphs with irreflexive links, are the driving force behind the methodologies. However, although the translation-based technique is the most used method for embedding, other methods are also used to simulate reflexive interactions.

### Multiplicative models

Multiplicative embedding model enable vectors to interact via dot products of entities. DistMult. HolE and Canonical decomposition models are applied on scholarly domain in literature. HolE models entity and relationship using circular correlation operator and captures asymmetric as well as anti-symmetric relations. A large scale knowledge graph, AceKG [106] is presented which attempts network representation learning based on five field of studies for scholar classification and clustering. Various additive (translation-based) and multiplicative embedding methods are applied to find missing links. However, holographic embedding HolE achieves most significant performance on anti-symmetric relations such as $field\_is\_part\_of$ and $paper\_is\_written\_by$. Furthermore, an application of embedding vector in scholarly domain is explored in [107] in which semantic structure is focused using canonical decomposition that uses complex embedding to handle asymmetry. A general framework to apply semantic queries such as analogy query and analogy browsing to solve exploration task is designed. In addition, various knowledge graph embedding models are employed on SKG [108] that gathers information relevant to the topic of social good. In order to create SKG, domain and topic conceptualization as well as data collection steps are performed. In this paper, anti-symmetric relations are handled using ComplEx with 93.66% hit rate and recommendations are computed based for the entities such as author, publication and Venue.

Another novel work [111] for knowledge completion is implemented on AIDA knowledge graph that incorporates a variant of DistMult. Two triple loss techniques weighted triple loss and rule loss are proposed and evaluated on DistMult embedding that outperformed various state-of-the-art embedding techniques. Though, DistMult is not suitable for asymmetric and anti-symmetric relations, it uses entry-wise product of head and tail entities. The score of triple*f*(*h*, *r*, *t*) and optimization framework is modeled as follows where $w_{h,r,t}$ is the weighted triple loss and $\eta ^{+^2}_{h,r,t}$ is the trainable variable.

$$\begin{aligned}&w_{h,r,t}-\eta ^{+^2}_{h,r,t} \le f(h,r,t) \le w_{h,r,t}+\eta ^{+^2}_{h,r,t}\\&\min _{\theta }\sum _{(h,r,t,w_{h,r,t})\in {(\tau _w)\cup {\mathcal {N}}}} \lambda _{1} \eta ^{-^2}_{h,r,t} + \lambda _{2}\eta ^{+^2}_{h,r,t}+ \lambda _{3}{\mathcal {L}} \end{aligned}$$

where $\lambda _1, \lambda _2$ are hyper-parameters that affect the degree to which trained variables are minimized whereas $\lambda _3$ is the multiplier of regularization term ${\mathcal {L}}$ over embedding of entities and relations. Similarly, for rule weighted loss ${\mathcal {R}}$ is modeled as:

$$\begin{aligned}&\min _{\theta }\sum _{(h,r,t,w_{h,r,t})\in {{\tau _w\cup {\mathcal {N}}}}} \lambda _{1} \eta ^{-^2}_{h,r,t}\\ {}&\quad + \lambda _{2}\eta ^{+^2}_{h,r,t}+ \lambda _{3}{\mathcal {L}}+ \lambda _{4} \sum ^{l}_{i=1} {\mathcal {R}}_i\\&\text {where}, {\mathcal {R}}=\max {(w_{q1}*\cdots * w_{qn}-f(q_{n+1}),0)}. \end{aligned}$$

To predict the DDI [58], authors implemented embedding techniques and baseline machine learning models are trained from which Conv-LSTM classifier outperformed on the application of ComplEx embedding model. Multiplicative models generate embeddings using product functions that capture pairwise relational patterns in all head and tail entities. Furthermore, these models manage complicated embeddings, and the product function increases the computing cost of the model as well.

### Deep learning models

Deep learning models such as convolutional neural networks are used to organize parameters into distinct layers and integrate them with the input data in order to recognize significant patterns to embed entities and relationships. An improvement is employed by integrating the existing medical knowledge graph with KG completion methods [109]. On the basis of ConvE, the ConvTransE model preserves the properties of translation, such as TransE between entities and relationships. Translational (TransE), semantic matching (Distmult and ComplEx) and neural network model (ConvE and ConvTransE) are applied to predict new treatment relations in biomedical entities and out of which ConvTransE outperformed. Similarly, ConvCN [110] is a citation recommendation method, uses an extension of ConvKB embedding algorithm to encode citation behavior in the citation network. ConvKB is extended in order to handle citation relations specifically. Two new relation vectors are introduced to represent the relationship between head and tail entities instead of single relation vector. Each entity $< v_h, v_t>$ and relation vector $< v_{rh}, v_{rt}>$ are concatenated row-wise and the absolute difference between $v_1$ and $v_2$ is calculated.

$$\begin{aligned}&f(h,r,t)= |v_1-v_2|\times W+b \\&L=\sum _{(h,r,t)\in \{ \text {KG}\cup \text {KG}'\}} \log ({1+ \exp (l_{(h,r,t)}}\cdot {f(h,r,t)})). \end{aligned}$$

In addition, before the fully-connected layer, an intermediary computation step is included to connect the dimensionally reduced representation with the fully-connected layer in order to determine the final score. Deep learning-based approaches utilized the unexplored features in various domain-specific scholarly data by reducing frequency variations. These models uses more than one convolution layers on input data resulting into feature map. Basic models concatenate the head and tail embedding, whereas others capture more interactions by performing additional convolution operations instead of convolutions on entities and relations.

### Discussion

Embedding-based knowledge graph completion is themethod that relays on the representation learning of triples to capture semantics. In the literature, three types of embedding methods such as translational embedding method and multiplicative and deep learning-based models are used. It has been observed that, translation-based models are the widely used in this domain. Many studies have applied TransE, TransH, TransR, TransD and proposed embedding method to present the performance of embedding methods a shown in Fig. 4. Besides, three types of evaluation methods have been used widely to as metric such as MRR, Precision, Hits.

One of the applications of Knowledge Graph Embedding models has been reported to give link predictions, which may also be viewed as a foundation for recommendation services. Embedding methods are applied to score triples to complete the knowledge graph by predicting the certain property. However, this service suffers from the challenge of sparsity in data due to insufficient interactions. Therefore, the link prediction task helps to improve the recommender system’s accuracy and diversity. This section deals with the link prediction problem where latent triple is given for some entities and relation and missing links need to be predicted. The identified links are proposed as collaboration recommendations analyzed the scientific profiles of the selected researchers from the domain-specific communities. Table 7 presents embedding methods that extract triples where paper and author are the head entity primarily whereas venue, author, field are the tail entity used to generate triple types. However, all the translation-based models depict entities solely on the basis of structural data, ignoring the richness of multi-source data contained in the entity’s name, description, category, relationship type and prior knowledge. Second, Neural network models have not gained much popularity in spite of gaining recognizable performance. CNN-based models such as ConvE embedded 2D convolution leads to long training time due to numerous parameters. Thus, more work should be performed in the direction of interpretability of predicted knowledge where small number of parameters are considered and non-expensive to use. In knowledge graph containing scholarly metadata, building recommendations of relevant collaborations is one of the important task. Most of the existing approaches for author collaboration focus on semantic similarities using bibliographic metadata such as publication counts and citation network analysis. However, these approaches abandon relevant metadata information such as author organization and venues attended, affecting the quality of the recommendations. In addition, the performances of existing models drop when they are applied as an embedding learner for entity typing in the task of scholar profiling. Studies should target to construct scholar profiles covering scholar’s research records and the popular domains that are highly relevant to them. Finally, one direction to pursue is developing unique approaches for understanding the interaction mechanism between multi-embedding vectors and their effective extension to subsequent embedding vectors.

**Table 7:** Summary of knowledge graph embeddings in scholarly domain

Embedding type (ET)	References	Applied ET	Triple $<h,r,t>$	Dataset	Task	Best performing ET	Evaluation metrics	Application
Translational	[98]	TransE, RotatE, ComplEx, Trans4E	$<paper, hasTopic, topic>$, $<paper, hasGRIDType, type>$	AIDA	Link Prediction	Trans4E	MRR, Hits	–
	[99]	TransE, TransH, TransR, TransD	$<Author, Publish, Paper>$,$<paper,belongto, venue>$,$<author, work, affiliation>$,$<paper, publishyear, year>$,$<paper, citation, paper>$	DBLP	Prediction	TransD	P, R, MRR	Paper Recommendation
	[100]	TransE, ComplEx, ConvE, RotatE, Trans-RS, TransE-SM and RotatE-SM	$<paper, hasAuthor, author>$, $<author, hasCoauthor,author>$, $<author/paper, hasVenue,venue>$	DBLP, semanticscholar, springernature, grid	Link Prediction	RotatE-SM	MRR, Hits	Author Recommendation
	[101]	TransE, RESCAL, TransH, TransR, TransD, TransP	$<scholar, verbphrase, organization>$	maui-semeval 2010	Entity Typing	TransP	P, R, F	Scholar Profile construction
	[102]	TransE, DBOW	–	DBLP, semanticscholar	Classification	TransE and DBOW combined	P, R, F	Paper Recommendation
	[103]	TransE, RotatE, DistMult, CompIE	$<Drug, TREATS, gene>,<Drug, INHIBITS, gene>, <Drug,INTERACTS_WITH, gene>$ and 12 others	PubMed and CORD-19	Link Prediction	TransE	MRR, Hits	Drug repurposing and to generate mechanistic explanations
	[104]	DKRL, DistMult, TransE, TransH, TransR	$<author, isCoAuthorOf, author>$, $<papers, isPublished, Event>$, $<Author, isAffiliatedin, departments>$	DBLP, semanticscholar, springernature, grid	Link Prediction	TransE	MR, Hits	Co-authorship Recommendation
	[105]	RotatE	$<Drug, Blocking, gene>,<Drug, treatment, disease>,<gene, binding, gene>,<gene, casualmutation, disease>$ and 35 others	PubMed	Link Prediction	RotatE	AUC	Recommending drug candidates for repurposing
	[99]	TransD, TransD, TransH, TransE	$<a, hasGridType, b>$, $<a, hasTopic, b>$	AIDA, MAG	Link Prediction	TransD	P, R, MRR, NDCG	–
	[59]	TransD, TransE, Distmult, and ComplEx, RotatE, Node2Vec	$<CHEMICAL, CHEMICAL-PROTEIN, PROTEIN>, <CHEMICAL, CHEMICAL-INDUCED-DISEASE, DISEASE>$	CORD-19	Link Prediction	TransD	P, ROC	Association analysis
Multiplicative	[106]	TransE, transH, DistMult, HolE, ComplEx	$<Paper, publish\_on, venue>$, $<paper, is\_in\_field, field>$, $<paper, is\_writtenby, author>$, $<authors, work\_in, institutes>$	AK18K	Link Prediction	HolE	MRR, Hits	Scholar classification and scholar clustering
	[107]	CP, Word2Vec	–	–	Data Exploration	–	–	Querying and Browsing
	[108]	TransE, TransD, TransR and ComplEx	$$<Author, authorOf, Paper>$$, $$<paper, belongsToDomain, Domain>$$, $$<Author, isCoauthor, Author>$$, $$ <Author, hasPaperIn, Venue>$$, $$<paper, isCitedBy, paper>$$, $$<paper, isPublishedIn, Venue>$$, $$<Author, isCitedBy, Author>$$	Collected using web crawler	Link Prediction	ComplEx	Mean Rank, Hits	Paper, author and venue recommendations
	[111]	TransE, Distmult,and ComplEx	$<b, hasPaper, a>,<b, hasEntityType, a>,<b, hasGridType, a>, <b, workedIn, a>$	AIDA35k	Link Prediction	WGE	MSE, MAE, F, Accuracy	Classifying research articles
	[58]	ComplEx, SimpleIE, TransE, CrossE, RDF2Vec	$<drug, hasTarget, protein>,<drug, hasTarget, gene>,<drug, hasEnzyme, protein>,<drug, hasEnzyme, gene>,<drug, hasTransporter, protein>,<drug,hasTransporter, gene>,<protein, isPresentIn, pathway>,<gene,isPresentIn, pathway>, <pathway, isImplicatedIn, phenotype>$	CORD-19	Link Prediction	ComplEx	AUPR, F	DrugDrug Interaction Prediction
Deep Learning	[109]	TransE, Distmult,and ComplEx, ConvE, ConvTransE	$<b, TREATS, a>, <b, IS\_TYPE, a>$	PubMed	Link Prediction	ConvTransE	Hits	Classifying drug candidates for repurposing
	[110]	ConvCN	$<citing paper, a citation type, a cited paper>$	Aminer	Link prediction	ConvCN	MRR and Hits	Citation Recommendation

**Fig. 5:**
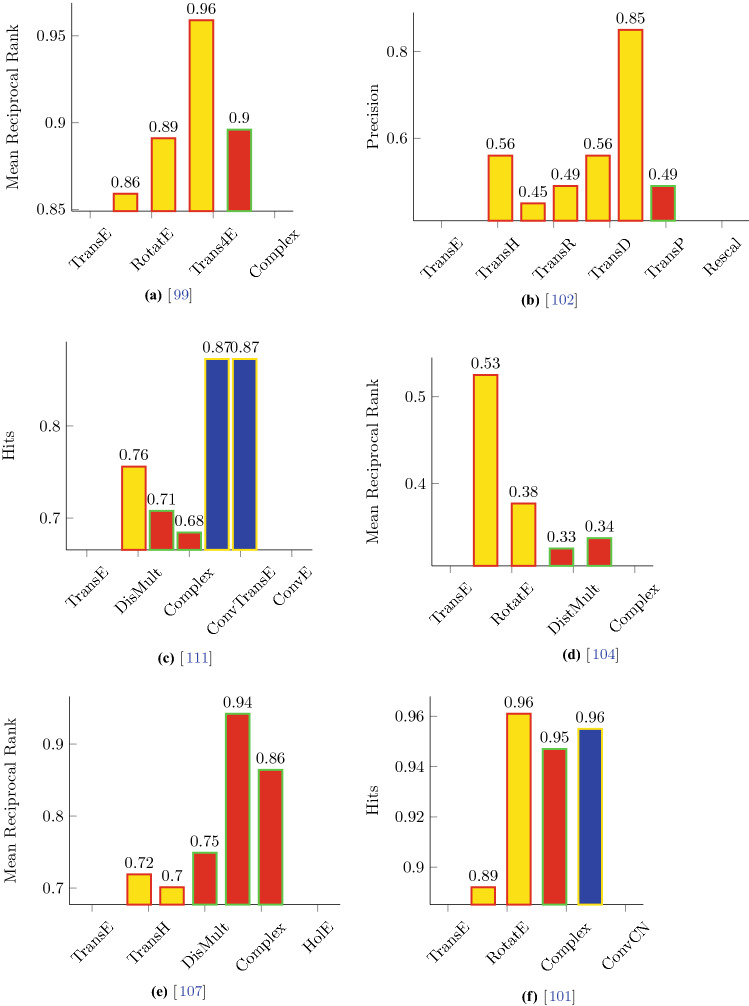
Comparison of papers based upon precision, hits and mean reciprocal rank

## Scholarly knowledge graph evaluation, ontologies, data models

**Evaluation:** During the construction of SKG, erroneous facts about entities and/or relationships may be collected. This technique is prone to errors, especially when using information collected from data sources of heterogeneous sources with variety of properties. During the process of evaluation, the reliability of the data source as well as the entire construction process of KG must be taken into account. In this survey, knowledge graph contains both ways of evaluation, one for quality of information extracted and quality of construction of knowledge graph. Information extraction evaluation includes quality about the concepts and their associations extracted along with the form of fact or triple. KG evaluation involves with the strategy to check the accuracy of the type of knowledge graph constructed. Although, there is no common standard evaluation protocol and set of benchmarks for the evaluation. It is difficult to construct a comparison standard that compares the evaluation methods based on their addressed criteria. However, three components of assessments are taken into account when assessing the overall quality of the knowledge graph.

- **Gold standard-based evaluation** This method involves with the comparison of designed KG with existing, manually annotated knowledge graph of the same domain. Matching domain-specific and autonomously generated KGs provides great significance in knowledge graph creation. With respect to evaluation methods, precision and recall are quite frequently used in information extraction as well as knowledge graph construction with machine learning methods. Other metrics, e.g., accuracy, area under curve (AUC), Hits@k and MRR, etc. are observed as better choice for evaluation during refinement. Furthermore, because a gold standard defines an ideal situation of collected concepts and constructed KG for a given domain, it is used to determine if the mapped information adequately covers the domain or whether it contains irrelevant domain-related elements. Applying gold standard, on the other hand, produces extremely accurate and reusable findings, but it is expensive to construct.
- ** Manual evaluation via domain experts and annotators** is the quality metric that usually predict accuracy with the agreement of the human annotators. This type of evaluation carries samples of results and allowed to apply for the detailed analysis of the approaches. In [59], two subset from data set are created to provide ratings by physicians to analyze relatedness of entities and to finalize embedding method. To evaluate the correctness (Is the information correct?) of the classification assigned to the concepts in NG-PL [52], subset of 1000 entities are annotated by six human annotators. Similarly, to evaluate the coverage (percentage of queries which can be answered by the knowledge graph) of the knowledge graph proposed approach is compared with baseline approach. Researchers should examine different data quality characteristics, such as relevance, completeness, modularity, conductivity, and so on, while developing the assessment techniques.
- ** Application-based evaluation via competency questions** which analyses the competency questions asked and likely to be answered by knowledge graph. Some studies, for example, conducted a casual and subjective evaluation with the help of survey questions and research questions [70, 74, 76] of the KG structure without using precise evaluation measures.

**Ontologies** Recent developments of intelligent knowledge base have heightened the need for semantic modeling to coordinate interactions of information systems. To improve the information unification, formation of ontological model and its integration is important for automating the process of implementing formal semantics. Ontology allows refinement of structure of knowledge and reduces conceptual ambiguity. The development and learning of ontology utilizes the description about many concepts and objects as well as relationships between them. In scholarly knowledge graphs, ontologies are the core elements that conceptualizes scientific semantic communication. All information is surrounded by entity types and relationships such as authors/researchers, articles, venue, domains, organizations, research problems, tasks, datasets, metrices and other artifacts.This objective is achieved by developing various ontologies to describe scholar’s artifacts. There are various conceptual models that are classified into groups from representation of specific research areas to describing structure of the scholarly documents, rhetorical elements and bibliographies [80, 112]. This category focuses on machine-readable representation of knowledge in scientific publications which expresses high semantic specifications.

SemSur (Semantic Survey Ontology) is a new ontology for modeling components of research contributions in the domain of Semantic Web. It is a comprehensive ontology for capturing the content of computer science articles and represent it in a semantic and machine interpretable format. It includes research problems, implementations used, and experiment setup and makes them more comparable. Aurora [38] utilized this ontology and explores the research findings in the articles based on an explicit semantic representation of the knowledge. Similarly, Computer Science Ontology [80] is an ontology for describing higher-level Computer Science study fields, as well as the sub-topics and words that go with them. This classifier powered numerous hybrid knowledge graphs [78, 79, 81] and explored by applications of KG also such as ResearchFlow [91]. A Friend Of A Friend (FOAF) ontology is used in [71, 73] to materialize implicit knowledge about the social relationships of authors and scientists. It is widely used ontology to explore properties related to social activities by integrating the related sources. In addition, Academia Industry Dynamics OWL schema is used that describes multifaceted information flow across academia and industry by integrating author’s affiliation and industrial sectors.

To fill the gap between domain-specific and semantic publishing ontology, Semantic Publishing and Referencing Ontology (SPAR) is widely used in various projects and publication such as [74]. In the literature, Software Ontology (SO) is used in [60] that extracts software mentions by employing neural network-based classifier in the scientific documents. Ontological representations permit knowledge to be semantically modeled in the concept of knowledge graphs. It is observed that quality evaluations of ontology is required to meet the criteria of construction of knowledge graph. **Scholarly Data Models** One of the features of Knowledge graphs is their emphasis on metadata, such as titles, abstracts, authors, and organization contained in research articles. Several notable projects are extracting knowledge about the prescribed metadata such as Microsoft Academic Graph, Aminer, ORKG and more. All of these efforts are aimed at providing tools and services for semantic analysis of scholarly themes, author networks, and bibliometric impact assessments, among other things.

- DBLP is based on AMiner’s citation network data set enriched with topics from the CSO Ontology using the CSO Classifier on paper abstracts.
- SciERC: Abstracts of 500 scientific articles from 12 distinct artificial intelligence conferences and workshops are available on SciERC. Abstract annotation is done by hand on five different places for each of the seven relationships.
- MAG: A heterogeneous and attributed knowledge graph containing the metadata of more than 242M scientific publications, including citations, authors, institutions, journals, conferences, and fields of study. It is a dynamic graph with evolving structure as new entities and relationships are added to the graph.
- MEDLINE: A bibliographic database covering various healthcare domains containing 12 million citations from 1960s.
- CORD-19: The COVID-19 Open Research Dataset (CORD-19) contains information about 63,000 research articles, related to COVID-19, SARS-CoV-2 and other similar corona viruses and from the Allen Institute for AI. The articles have been collected from various scientific corpus such as bioRxiv, medRxiv, and PubMed Central
- PUBMED: A combination of PubMed and non-PubMed data sources from medicine, health care systems, clinical sciences and PubMed Central. Various scholarly knowledge graphs have built their own datasets by crawling data from various digital libraries, including Web Of Science, GRID, PharmaGKB, Dimensions among others.

## Scientific knowledge graph application/tasks

- Open IE and KG: In NLP, traditionally information extraction techniques incline to use a predefined set of target schema that contains an agreed set of specific concept and relation types for building knowledge graphs. Unlike conventional IE technique, Open Information Extraction (OIE) is a way to generate machine readable though domain-independent representation of information in the form of triples and proposition. OIE models relay on unsupervised information extraction techniques and pre-trained on heterogeneous datasets. It focuses on smaller but denser corpora rather than bigger and sparse corpora. Open information extraction techniques make use of a set of patterns to extract triples consisting of two arguments, a subject, an object and a predicate (relation) linking the arguments, which can then be used to construct a knowledge graph. It works towards the improvement of recall for better coverage in order to discover new attributes.
- Recommendation and ranking service: In the literature, knowledge graphs are integrated as an information source to improve recommendations and inherently provides more interpretability in knowledge representation. Recommendation can be interpreted as a knowledge graph completion problem where various translational and semantic matching-based embedding methods outperformed. Scholarly knowledge graph provides services such as intelligent contextual recommendation and ranking [113, 114] by discovering information from the scientific articles. To provide recommendations for scholarly networks using knowledge graphs explores not only explicit but also implicit relationships. Second, multiple resources may also be consider to construct multidimensional recommendations effectively. For example, WoS [115] presented a knowledge graph-based system to extract and rank scholar’s profile as well as represents relationships among scholars. A new explicit ranking scheme [113] is proposed that models relatedness of query entity and document entity using the exact match and soft match signals. In this paper, an academic knowledge graph is constructed using semantic scholar’s query log and explored soft match using knowledge graph is effective while word-based ranking models capture the semantic meaning unsuccessfully. A paper recommendation in [82] analyzed topic similarity, citation similarity to show links between paper nodes using semantic, KGE and relational GCN approaches. A very important work by [116] for method recommendation is performed by applying semi-supervised approaches to explore multiple relations. In order to reduce efforts for human annotation task, term co-occurrence and dependency paths are explored and scientific recommendations are produced. To best of our knowledge, certain filtering issues such as sparsity, diversity and cold-start have not been taken into account.
- Explainable scholarly knowledge graphs: Graph-based knowledge representation involves with querying and reasoning mechanisms for transparent and (human and machine) interpretable explanations [103]. To understand inferences of information, ascertaining significance of an entity is critical using linked data and ontologies. In this view, a central challenge of consistent knowledge matching is evolved in case of manual and automated construction of scholarly knowledge graphs. Mining (classifying and clustering) of scholarly entities and relationships, question answering with trust and scientific fact-checking explanations [117] are worth mentioning problems to claim the scope of Explainable AI (XAI) with scholarly knowledge graphs. Through tracing over KG, the XAI system assists stakeholders in conceptually understanding the workings of associated systems in order to achieve explainable outcomes and interpretability. For example, domain knowledge infusion model helps to explain author’s impact by tracing the author’s research history and derived impact’s explanations can serve as a platform for recommendation.
- Scientific Question Answering: Transformations from normal text-based search engines to a question–answer service with semantic awareness is a very crucial task. Understanding of relationships between input query and supporting content is very important phase in this knowledge extensive task. [88] Proposed a computational linguistics knowledge graph (CLKG) that is used to crawl metadata (article, author, venue, field) for entity-specific query retrieval framework. In addition, JarvisQA [118] is a BERT-based question–answer system that retrieves answers from variety of tables via Table2Text converter. [119] explores the power of scigraph for questioning answering. [68] developed a question–answer framework to retrieve answers from background corpora that integrates knowledge graph matching and semantic matching using BioBert language model.
- Academic mining and author disambiguation: Research Group Knowledge Graph [120], Veto [121], automatic evidence mining [122], finding rising stars, automatic paper draft generation are few applications possessing academic mining as well as background of knowledge graphs. Second, author is an important entity in SKG and disambiguation [61] of this particular entity is one of the intensive research interest. Lack of a unique normalized identity of an author entity makes the problem more challenging for certain services such as expert finding and collaborator search. For example, two authors may have similar name, affiliation and title. In such case, identification of described entity in large-scale system can be complex in order to process a name-based query.

## Future directions/challenges

- Heterogeneity and Linking of research objects: Extraction of structured knowledge is a challenge across the board and one of the reason for this is data ingestion from multiple resources which makes the knowledge noisy and inconsistent. Integration of information from heterogeneous sources can cause labor-intensive human annotations to train knowledge extraction systems. This can be reduced by adopting fully unsupervised approaches as compared to traditional supervised machine learning approaches. Maintaining heterogeneity along with embedding in order to map links into low dimensional order is a great challenge. For example, integrating through social networks may cause inconsistent set of triplets due to significant unstructured information. The experts in the field of knowledge graphs have merely illustrated the potential applications and deep insights in the field of network analysis, community detection, retrieving neighbors and advanced clustering. The level of data integration is immature and fragmented due to redundancy till now. Second, a unique and persistent identifier is required to identify the relevant digital objects that possess human-readable label feature. In the practice, identifiers helps in distilling specific information and provides machine-actionable metadata to the research communities using information systems.
- Generation of FAIR Literature surveys: To view the quality aspect of the work, FAIR guiding principle (Findable, Accessible, Interoperable, Reusable) [123] is the important scientific merit for Scientific knowledge graphs. The scientific information provided in the literature, on the other hand, does not fulfill the FAIR Data Principles. Because of the publishing style, components of literature surveys, such as survey tables published in scientific publications, do not conform to the FAIR criteria. It is critical to follow the FAIR principles and contribute significantly to baseline review reuse and their enclosed information. Generating FAIR literature surveys [27], FAIR-compliant research contribution model [54], transforming data set into knowledge graph by following FAIR data principles [74, 124]is important to achieve for better quality.
- Ontology matching: The design of ontology to conceptualize and model scholarly knowledge to enable its exchange across different SKGs is important. Many scholarly ontologies are restricted over certain domain-specific entity sets. Another issue is that researchers seeking relevant information have to deal with multiple data sources as well as unstructured search. Ontologies underlying the knowledge graphs possess this issue of information foraging and required to reduce the cost associated with database scenario and textual search. Matching semantic set of properties and determining the similarity of resources is one of the important subtask.
- Knowledge extraction from diversely structured textual data: Many studies pays attention to the extraction from structured or semi-structured data sources. Studies based on knowledge extraction from unstructured data sources such as images, tables and pseudocode of an algorithm is limited to date. In order to obtain an overall machine-actionable scholarly knowledge graph, aligned resources are required that help achieve a cutting-edge standard [125] to model scholarly disciplines. For example, table metadata extraction [126] possess diverse challenges due to lack of standardization. In order to extract and characterize them in a machine-readable representation layout and cell-content metadata are required to design flexibly. In [127] model is prepared for customized chart visualizations from tables to provide more detailed overview of context. To provide simplification and standardization, nanopublications [128], i.e., a fine-grained, machine-interpretable, semantic and interlinked representation for article information (sections, text, figures, tables, formula, footnote and review comments), provenance and assertions is presented as an RDF graph. To model multiple knowledge graphs considering multiple components such as text, images and source code, [129] from deep learning papers is constructed and led to an aggregated knowledge graph. Similarly, Dia2Graph performed diagram extraction, classification and graph generation from deep learning diagrams.
- Quality assessment and evaluation: Mediating the quality of algorithmic outputs produced by knowledge graph construction module is a challenging task. It is improbable that involved algorithms will be evaluated based on human designed gold standard and human annotators in prospecting years. A major challenge is to be sure about the goodness of algorithm in terms of verification and validation.
- Completion of the knowledge graph: Metadata used in construction of knowledge graphs suffers from data incompleteness to different degrees such as affiliation ambiguity. Similarly, identify and incomplete references, author disambiguation and citation count mismatch tends to vary on different metadata.

## Conclusion

In recent time, knowledge graphs have been emerged as the illustration of many real-time applications and implemented practically to classify entities and relationships. In this context, knowledge graph in scholarly domain is the specific area in which semantic representation of literature-based discovery is presented. In this paper, we presented a broad and accessible introduction with relevant directions about scholarly knowledge graphs and discussed common infrastructures of graphs in scholarly domain. Various potential implementations using machine learning approaches, natural Language processing approaches, rule-based reasoning and hybrid approaches are described. Issues in integration of data sources, ontology matching, extracting KG from diversely structured documents, cross-domain scholarly KG are identified as the future work through the survey. A detailed analysis of applications is also explained from different perspectives like scientific question answering, recommendation service, Open Information Extraction along with their potential challenges. Overall, we are able to conclude that knowledge graph is an important advancement and have power to provide semantically structured information to huge scholarly domain. However, efforts of applying such concepts into specific domains have been made in recent years, several aspects remain to be explored.
